# Human *ARHGEF9* intellectual disability syndrome is phenocopied by a mutation that disrupts collybistin binding to the GABA_A_ receptor α2 subunit

**DOI:** 10.1038/s41380-022-01468-z

**Published:** 2022-02-15

**Authors:** Dustin J. Hines, April Contreras, Betsua Garcia, Jeffrey S. Barker, Austin J. Boren, Christelle Moufawad El Achkar, Stephen J. Moss, Rochelle M. Hines

**Affiliations:** 1 Department of Psychology, University of Nevada Las Vegas, Las Vegas, NV USA; 2 Department of Neurology, Boston Children’s Hospital, Boston, MA USA; 3 Department of Neuroscience, Tufts University School of Medicine, Boston, MA USA

**Keywords:** Neuroscience, Autism spectrum disorders, Genetics

## Abstract

Intellectual disability (ID) is a common neurodevelopmental disorder that can arise from genetic mutations ranging from trisomy to single nucleotide polymorphism. Mutations in a growing number of single genes have been identified as causative in ID, including *ARHGEF9*. Evaluation of 41 *ARHGEF9* patient reports shows ubiquitous inclusion of ID, along with other frequently reported symptoms of epilepsy, abnormal baseline EEG activity, behavioral symptoms, and sleep disturbances. *ARHGEF9* codes for the Cdc42 Guanine Nucleotide Exchange Factor 9 collybistin (Cb), a known regulator of inhibitory synapse function via direct interaction with the adhesion molecule neuroligin-2 and the α2 subunit of GABA_A_ receptors. We mutate the Cb binding motif within the large intracellular loop of α2 replacing it with the binding motif for gephyrin from the α1 subunit (*Gabra2*-1). The *Gabra2*-1 mutation causes a strong downregulation of Cb expression, particularly at cholecystokinin basket cell inhibitory synapses. *Gabra2*-1 mice have deficits in working and recognition memory, as well as hyperactivity, anxiety, and reduced social preference, recapitulating the frequently reported features of *ARHGEF9* patients. *Gabra2*-1 mice also have spontaneous seizures during postnatal development which can lead to mortality, and baseline abnormalities in low-frequency wavelengths of the EEG. EEG abnormalities are vigilance state-specific and manifest as sleep disturbance including increased time in wake and a loss of free-running rhythmicity in the absence of light as zeitgeber. *Gabra2*-1 mice phenocopy multiple features of human *ARHGEF9* mutation, and reveal α2 subunit-containing GABA_A_ receptors as a druggable target for treatment of this complex ID syndrome.

## Introduction

Intellectual disability (ID) is a common neurodevelopmental disorder that can co-occur with one or more clinical co-morbidities that together comprise a syndrome. Genetic mutations are a common underlying cause of syndromic ID, with trisomy 21 (Down syndrome) being the most prevalent [1]. In addition to chromosomal abnormalities affecting numerous genes, a growing number of single genes have been identified as causative in ID syndromes [2, 3], including *ARHGEF9* (Cdc42 Guanine Nucleotide Exchange Factor 9) [4]. *ARHGEF9* is located on the X-chromosome and as such is part of a larger group of X-linked ID syndromes, which account for approximately 10–12% of all cases of ID. To date, a relatively large number of different mutations in *ARHGEF9* have been identified as causative in a seemingly heterogenous syndrome characterized not only by ID, but also a high incidence of epilepsy and a number of other features with varying presentation [2, 4–23].

**Fig. 1:**
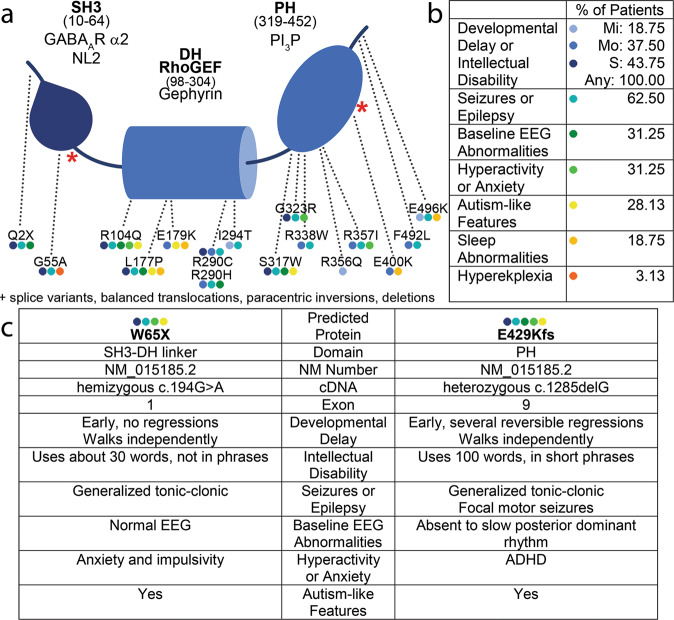
*ARHGEF9* mutations overlaid on collybistin protein structure, and relationship to the core phenotypes of the human *ARHGEF9* mutation syndrome. **a** Schematic diagram of Cb protein structure with reported point mutation sites leading to missense and nonsense mutations marked (new patients red asterisks). In addition to the 18 point mutations identified to date, the human *ARHGEF9* ID syndrome has also been linked to splice variants, balanced translocations, paracentric inversions, and deletions. **b** Listing of the core reported phenotypes of the human *ARHGEF9* mutation ID syndrome, and the proportion of patients reported to show each phenotype. **c** Clinical summary of the two patients characterized in the context of this study. Abbreviations: Src homology 3 (SH3) domain; Dbl homology (DH) domain; Rho Guanine Nucleotide Exchange Factor (RhoGEF); Plecstrin homology (PH) domain; neuroligin-2 (NL2); Phosphatidylinositol 3-phosphate (PI_3_P); mild (Mi); moderate (Mo); severe (S); electroencephalogram (EEG).

The *ARHGEF9* gene encodes a protein known as collybistin (Cb), which is a guanine nucleotide exchange factor (GEF) known to be an important organizer at synapses controlled by the neurotransmitters γ-amino butyric acid (GABA) and glycine [9, 24–26]. Cb is known to interact with phosphoinositides via its plextrin homology (PH) domain [27], and the c-terminus of the adhesion molecule neuroligin-2 (NL2) via its Src homology (SH) 3 domain [28] (Fig. 1A). Cb has recently been shown to directly interact with the α2 subunit of GABA_A_ receptors (GABA_A_Rs) with micromolar affinity, an interaction mediated by the SH3 domain of Cb and 13 amino acids within the large intracellular loop of the α2 subunit [29] (Fig. 1A). By contrast, the classic inhibitory synaptic organizer gephyrin has a stronger interaction with the α1/3 subunits of GABA_A_Rs [30], but relatively weak binding with α2 [29]. The α1 and α2 subunits also differ based upon their subcellular localization to inhibitory synapse subtypes, with α1 containing receptors being enriched at dendritic inhibitory synapses and at somatic synapses arising from parvalbumin (PV) positive basket cells, while those containing α2 are enriched at somatic synapses arising from cholecystokinin (CCK) positive basket cells and axon initial segment synapses arising from chandelier cells [31–33].

In the present study, we carefully examine published case reports of patients with *ARHGEF9* mutation for the incidence and prevalence of phenotypic traits. We find that in addition to ID and epilepsy, behavioral symptoms, baseline EEG abnormalities, and sleep dysregulation are frequently reported as components of this syndrome. In some of the clinical cases, these phenotypes are not necessarily absent but have not yet been reported. We next interrogate the utility of mice with a mutation in the Cb binding site within the GABA_A_R α2 subunit (*Gabra2*-1 mice) as a model for the human *ARHGEF9* mutation syndrome. We demonstrate that downregulation of Cb is a central effect of the *Gabra2*-1 mutation, critically linking the α2 subunit of GABA_A_Rs and Cb as partners. Cb cluster density and size are both decreased in *Gabra2*-1 mice, with a particular influence on CCK basket cell synapses, as evidenced by reduced colocalization of Cb with both CCK and Cannabinoid type 1 receptors (CB1Rs). *Gabra2*-1 mice are characterized by deficits in working and recognition memory indicative of ID, and also display anxiety-like behaviors and impaired social interaction. During postnatal development, a subset of *Gabra2*-1 mice are observed to have spontaneous seizures and are susceptible to mortality. We also find that the *Gabra2*-1 mutation results in a striking increase in baseline low-frequency EEG power that is maintained across the 24-hour period. Spectral analysis of specific vigilance states demonstrates that the increase in EEG power is significant in the δ frequency range during the state of non-rapid eye movement (NREM) sleep, and the θ frequency range during wake (W) and rapid eye movement (REM) sleep. Interestingly, this manifests as a reduction in time spent asleep during subjective night demonstrated using both behavioral and EEG measures. Further, *Gabra2*-1 mice show reduced transitions into and out of specific arousal states, particularly when transitioning between NREM and REM. *Gabra2*-1 mice also have an inability to maintain free running circadian rhythmicity in the absence of light as a zeitgeber. The phenocopy of multiple features of the human *ARHGEF9* mutation syndrome in *Gabra2*-1 mice suggests that the Cb-α2 subunit partnership is central to the phenotype. These results also suggest that in addition to ID and epilepsy, baseline EEG abnormalities, behavioral symptoms, and sleep disfunction may be considered as core features of the human *ARHGEF9* syndrome. This information may be used to inform or refine clinical diagnosis, as well as aide in the development of therapies used to alleviate multiple symptoms of the human *ARHGEF9* mutation syndrome by targeting α2 subunit-containing GABA_A_Rs.

## Materials & Methods

### Clinical cases

The protocol for obtaining phenotype information on patients was approved by the Institutional Research Board at Boston Children’s Hospital/Harvard Medical School. Informed consent for participation in this research was obtained from both families.

### Study design

The study design was based on the ARRIVE guidelines (Animal Research: Reporting of In Vivo Experiments) [34], and additional details can be found in the supplementary methods. Studies were conducted by experimenters and observers blinded to the genotype of the animal, with the exception of western blotting where genotype was known to organize loading of samples. The *Gabra2*-1 strain is congenic on a C57Bl6 J background, and cohorts composed of *Gabra2*-1 homozygotes and wildtype (WT) littermate controls were used for all experiments. Littermate groups originated from two to four litters from distinct parental mating pairs, and were arranged into cohorts containing at least 2 animals of each genotype. Due to the need for genotype matching, animals were not randomized into groups. Animals within a cohort were tested in the same day, and additional cohorts were tested at approximately the same time of day, on a subsequent day as required. For mortality and spontaneous seizure studies, as well as western blotting and confocal microscopy experiments, both male and female offspring were used. In these studies, sex was examined as a biological variable (enabled by retrospective SRY PCR to determine sex in pups), with no effect of sex being found in the analysis. EEG and behavioral assessments were conducted on coded *Gabra2*-1 and WT mice in cohorts of males. All of the data that supports the findings of this study have been deposited in an institutional repository, and are available from the corresponding author upon reasonable request. Unique materials, including the *Gabra2*-1 mice are also available from the corresponding author upon request.

### Mouse generation and maintenance

Animals were cared for according to the NIH Guide for the Care and Use of Laboratory Animals and protocols were approved by the Institutional Animal Care and Use Committee (IACUC) of Tufts University School of Medicine or the IACUC of the University of Nevada Las Vegas as detailed in the supplementary methods.

### Western Blotting and Immunohistochemistry

Western blotting and immunohistochemistry were performed according to our established protocols, and are detailed in the supplementary methods. Antibodies used are detailed in Supplementary Table 7.

### Behavioral analysis

We conducted a basic phenotype screen using the modified SHIRPA protocol established by the European Mouse Phenotyping Resource of Standardized Screens (EMPReSS). Spontaneous alternation was tested in a T-maze and began with placing animals in the start arm of the maze for free exploration following the protocol by Deacon et al [35, 36]. The novel object recognition test was based on the protocol by Leger et al [37]. The light-dark exploration test, elevated plus maze, and the three chambered social paradigm were based on protocols from the Crawley lab and our prior work [38–40]. To analyze activity under normal diurnal and constant darkness conditions, animals were individually housed in home cages equipped with photobeam frames (TSE Systems), housed within a sound-attenuating chamber. Details of behavioral assessments are in the supplementary methods.

### Electroencephalography

Electroencephalography (EEG) and electromyography (EMG) recordings were performed as previously described [29, 36]. The details of the implantation, recording, scoring, and analysis can be found in the supplementary methods.

### Statistical analysis

Sample sizes were predicted based on power analyses run on pools of data from prior genetic modification experiments (with *n* = 4 animals per group yielding a power ≥80% on EEG experiments; *n* = 6 animals per group yielding a power ≥80% for immunohistochemical analysis of inhibitory synaptic puncta; *n* > 6 animals per group needed to yield a power ≥80% for behavioral experiments). No animals or samples were excluded from the study. Following experimentation, comparisons between genotypes were validated by statistical analysis using non-parametric tests (Kruskal–Wallis for multiple comparisons) as with Tukey Post Hoc as appropriate. Parametric tests were applied only after having validated the data with normality tests, and parametric tests applied include *t*-test; or one-way ANOVA, or repeated-measures ANOVA, with Bonferroni Post Hoc as appropriate. Significance was set at ≤0.05. Graphs are plotted as mean and standard error for bar and line graphs; or as median, first and third quartile, and range for box plots. A table summarizing statistical analyses is shown in the supplemental material (Supplementary Table 6).

## Results

### The human *ARHGEF9* intellectual disability syndrome is complex and partially heterogeneous

In prior studies, mutation in *ARHGEF9* has been reported to be the cause of ID syndromes in 39 patients [2, 4–23]. In the context of the present study, we examined two new patients with de novo mutations in *ARHGEF9* (Fig. 1a, c) that meet criteria to be classified as pathogenic variants [41]. The growing number of reports suggests that *ARHGEF9* mutation may be among the more common forms of X-linked ID [4]. Patient 1 (male, 16 years of age) was found to have a missense mutation in exon 1 (c.194 G > A; p.W65X), while patient 2 (female, 16 years of age) was found to have a missense mutation in exon 9 leading to frameshift (c.1285delG, p.E429Kfs; Fig. 1a, c). Both patients have ID, including limited use of language, as well as generalized tonic-clonic seizures with onset within the first two years of life (Fig. 1a, c). Seizures experienced by patient 1 are partially responsive to medications and are currently controlled, while the seizures of patient 2 have been unresponsive or only partially responsive to multiple medications (Supplementary Table 1). Both patients had early developmental delay, and meet criteria for diagnosis with Autism spectrum disorder. Patient 1 has additional behavioral symptoms of impulsivity and anxiety, while patient 2 has hyperactivity (ADHD).

Of the 41 total patients identified thus far, 28 are male and 13 are female, with skewing in favor of the abnormal X-chromosome common among the female patients [6, 11, 14, 17] (Supplementary Tables 2, 4). Across the 41 patients, 33 different mutations have been identified in *ARHGEF9*, including micro, partial, and full deletions, missense and nonsense mutations, balanced translocations, paracentric inversions, and splice variants (Fig. 1a and Supplementary Tables 2, 4) demonstrating the general instability of this portion of the genome. Missense mutations have been the most frequently reported (51.2% of *ARHGEF9* mutations; Fig. 1a and Supplementary Table 4), followed by deletions (31.7% of mutations). Severe ID is prevalent among patients with *ARHGEF9* mutation (46.3%), and all patients bear some extent of ID (and/or developmental delay (DD) in infants; Fig. 1a, b and Supplementary Tables 2, 3). Epilepsy is noted in the majority of cases (63.4%) of the human *ARHGEF9* mutation syndrome reported to date, and abnormalities in the baseline EEG (31.7%) are also commonly reported (Fig. 1a, b and Supplementary Tables 2, 3). Epilepsy appears to be correlated with ID in *ARHGEF9* mutation syndrome, being common among those individuals characterized by moderate to severe ID^4^. The baseline EEG abnormalities involve primarily the low frequency spectral wavelengths including δ and θ bands. Behavioral symptoms of anxiety, hyperactivity, and autism-like features have been noted in many patients (Fig. 1a, b and Supplementary Tables 2, 3). Of interest, sleep disturbances were also frequently documented in *ARHGEF9* mutation patient reports (Fig. 1a, b and Supplementary Tables 2, 3), while other case studies did not report on the presence or absence of sleep symptoms in the patients described. Thus, sleep disturbance may be an additional dimension worthy of evaluation in *ARHGEF9* mutation. Taking together the new and previously documented cases, patterns are emerging in the constellation of symptoms that accompany ID in the human *ARHGEF9* mutation syndrome, all of which are indicative of alterations in the patterning of cortical activity and suggestive of impaired cortical inhibition.

### Mutating the large intracellular loop of the GABA_A_R α2 subunit strongly downregulates collybistin expression at subtypes inhibitory synapses

**Fig. 2:**
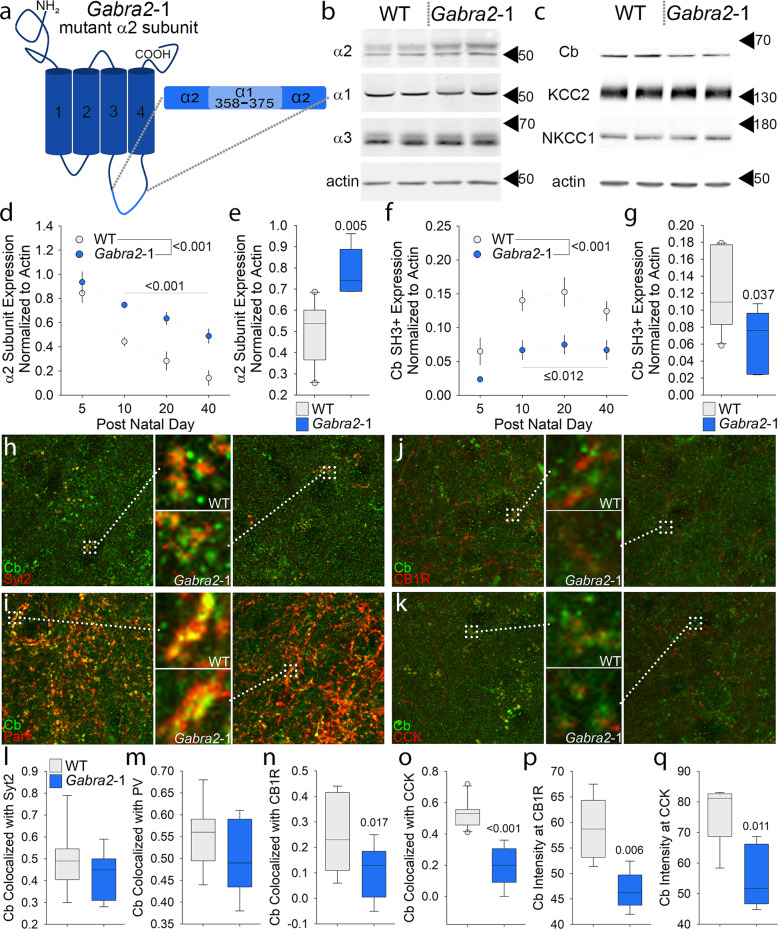
A mouse model with a mutation in the GABA_A_ receptor α2 subunit large intracellular loop (*Gabra2*-1) strongly influences collybistin expression at specific subtypes of inhibitory synapses. **a** Schematic diagram of the mutation generated in the large intracellular loop of the GABA_A_ receptor α2 subunit large intracellular loop, at the site where Cb is characterized to interact. The Cb interaction motif has been replaced with a motif from the α1 subunit that preferentially interacts with gephyrin generating the *Gabra2*-1 mouse. **b** Western blotting of cortical lysate from PND 10 suggests that the *Gabra2*-1 mutation increases the total expression of the α2 subunit, but does not change expression of either α1 or α3 subunits. **c** Western blotting of cortical lysate from PND 40 suggests that the *Gabra2*-1 mutation increases the total expression of Cb. Expression of total KCC2 and NKCC1 appears unaltered by the *Gabra2*-1 mutation. **d** Quantification of changes in total α2 subunit expression across a time course of postnatal development in both WT and *Gabra2*-1 cortical lysates. **e** Quantification of α2 subunit expression from PND10 comparing WT and *Gabra2*-1. **f** Quantification of changes in total Cb expression across a time course of postnatal development in both WT and *Gabra2*-1 cortical lysates. **g** Quantification of Cb expression from PND40 comparing WT and *Gabra2*-1. **h** Immunostaining for Cb and Syt2 in WT and *Gabra2*-1 mice show closely opposed clusters on the soma of cortical cells. **i** Immunostaining for Cb and PV in WT and *Gabra2*-1 mice show colocalized clusters on the soma of cortical cells. **j** Immunostaining for Cb and CB1R in WT mice show closely opposed clusters on the soma of cortical cells. **k** Immunostaining for Cb and CCK in WT mice show closely opposed clusters on the soma of cortical cells. **l, m**. Quantification of colocalization (Pearson’s) of Cb with Syt2 (l) and PV (m) shows no change between WT and *Gabra2*-1. Colocalization (Pearson’s) of Cb with both CB1R (**n**) and CCK (**o**) is reduced on the soma of *Gabra2*-1 cortical cells. Quantification of Cb intensity at CB1R (**p**) and CCK (**q**) positive clusters on the soma of cortical cells reveals a significant reduction in Gabra2-1. Graphs d,f plot mean and standard error; graphs **e**, **g**, **l**-**q** plot median, first and third quartile, and range.

*ARHGEF9* encodes the protein Cb, which is an important organizer at inhibitory synapses [24–26], known to interact with the α2 subunit of GABA_A_R’s [29] among other inhibitory synaptic proteins [28]. Our previous work has shown that 13 amino acids within the large intracellular loop of α2 interacts with the Cb SH3 domain with micromolar affinity [29]. Based on this interaction, we generated *Gabra2*-1 mice that express a 13 amino acid substitution from the α1 subunit into the α2 subunit large intracellular loop, effectively replacing the Cb binding sequence with the gephyrin binding sequence [29] (Fig. 2a). Total α2 subunit expression is enhanced by the *Gabra2*-1 mutation, evident by postnatal day (PND) 10 and persisting into maturity (Fig. 2b, d, e). α1 and α3 subunit expression is not significantly altered at any point along the postnatal developmental time course (Fig. 2b and Supplementary Fig. 1a, c, d, h, i), nor was expression of the ion co-transporters KCC2 (K + Cl− type 2) and NKCC1 (Na+ K + Cl− type 1; Fig. 2c and Supplementary Fig. 1b, f, g, j, k). Of particular interest, total Cb expression is strongly downregulated in *Gabra2*-1 mice, evident by PND 5 and persisting into maturity (Fig. 2c, f, g and Supplementary Fig. 1a), while total gephyrin [29] and NL2 (Supplementary Fig. 1b, e) remain unchanged.

Immunohistochemistry for Cb demonstrates that cluster size and density is dramatically decreased in samples from the cortex of *Gabra2*-1 mice compared to WT controls (Supplementary Fig. 2a). Of interest, Cb positive clusters that remain in *Gabra2*-1 cortex still colocalize with both synaptotagmin 2 (Syt2) [42] and PV clusters on the soma (Fig. 2h, i, l, m and Supplementary Fig. 2b, c), suggesting that Cb localization at synapses arising from PV-positive basket cells remains unaltered in *Gabra2*-1 mice. In contrast, we found that colocalization of Cb with both CB1R and CCK clusters on the soma was reduced (Fig. 2j, k, n, o), which was associated with a reduction in the intensity of Cb at CB1R and CCK clusters on the soma (Fig. 2p, q). These findings suggest that somatic inhibition arising from CCK-positive basket cells may be compromised in *Gabra2*-1 mice. This data suggests that the *Gabra2*-1 mutation strongly influences the extent of expression of Cb, and may have a particular influence on subtypes of inhibitory synapses that are normally enriched with the α2 subunit.

### *Gabra2*-1 mice exhibit features of intellectual disability along with other behavioral phenotypes present in *ARHGEF9* mutation

**Fig. 3:**
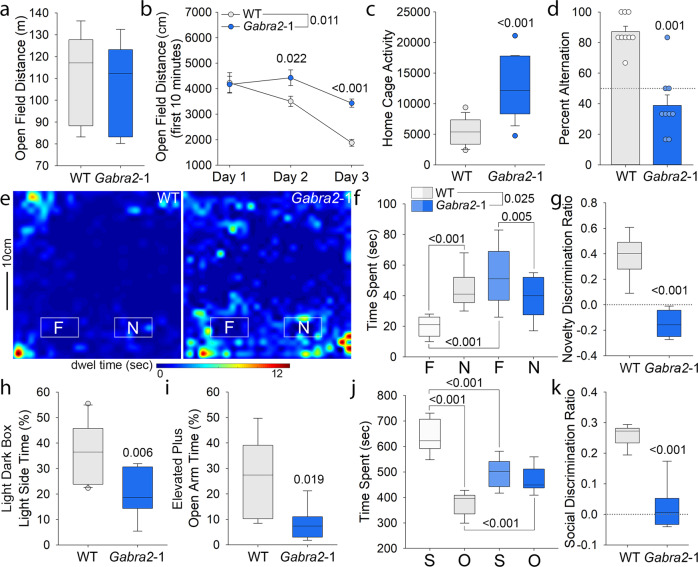
*Gabra2*-1 mice have behavioral impairments that model intellectual disability as well as other features of *ARHGEF9* mutation. **a** *Gabra2*-1 mice have normal levels of distance travelled in the open field, suggesting adequate motor performance. **b** *Gabra2*-1 mice maintain high levels of activity across repeated exposures to the open field, suggesting that they do not recall their prior experience in the open field. **c** Assessment of home cage activity comparing single housed WT and *Gabra2*-1 mice is suggestive of hyperactivity in *Gabra2*-1. **d** *Gabra2*-1 mice fail to spontaneously alternate arm choice in the T-maze, indicative of working memory impairment. **e** Heat maps of exploratory activity during the test session of the novel object recognition paradigm, showing representative exploration of the familiar (F) and novel (N) objects by WT and *Gabra2*-1 mice. **f** Analysis of time spent exploring the F and N objects during the novel object recognition test session. WT mice spend significantly more time with the N object, while *Gabra2*-1 mice do not. **g** Analysis of the discrimination ratio for the N object, which is positive for WT mice, but negative for *Gabra2*-1 mice. **h** *Gabra2*-1 mice spend significantly less time in the light side of the light-dark box. **i** In the elevated plus maze, *Gabra2*-1 mice spend less time in the open arms. **j** Analysis of time spent with novel social (S) or object (O) stimuli in the 3-chambered maze by WT and *Gabra2*-1 mice. WT mice spend significantly more time with the S stimulus, while *Gabra2*-1 do not. **k** Analysis of the discrimination ratio for the S stimulus, which is positive for WT mice, but neutral for *Gabra2*-1. Graphs b,d plot mean and standard error; graphs **a**, **c**, **f**–**k** plot median, first and third quartile, and range.

ID is at the core of the *ARHGEF9* mutation syndrome, present in all of the cases reported to date. Thus, we next investigated whether the *Gabra2*-1 model has ID relevant phenotypes. Working memory is broadly found to be impaired in syndromic ID, including Fragile X and Down syndromes [1, 43, 44], and ID syndromes also feature impairments in episodic memory [1, 45]. We first examined open field behavior and found that *Gabra2*-1 show similar distance travelled and average speed of travel in the open field (Fig. 3a and Supplementary Fig. 3a–d). *Gabra2*-1 mice also show a similar habituation curve in response to the novelty of the open field during the first trial (Supplementary Fig. 3a, c). In subsequent open field trials, WT mice show a typical decrease in exploratory activity during the first 10 minutes (Supplementary Fig. 3e), while *Gabra2*-1 mice show a high level of exploration during the first 10 min across three successive trials on three successive days (Supplementary Fig. 3f). We next used spontaneous alternation with short intertrial intervals in a T-shaped maze in order to test working memory [35]. WT mice frequently alternate arm choice in the T maze, while *Gabra2*-1 mice have a level of arm alternation near chance (Fig. 3d). The low level of spontaneous alternation in *Gabra2*-1 mice suggests a deficit in working memory, which is needed to recall their last exploration of the T maze. In the novel object recognition paradigm [37] WT and *Gabra2*-1 mice significantly differ based on time spent with the novel object (Fig. 3e–g). During the test session, WT mice spend significantly more time with the novel (N) object, resulting in a positive discrimination ratio (Fig. 3g). *Gabra2*-1 mice spend significantly more time with the familiar (F) object in comparison to the novel (N) object, yielding a negative discrimination index (Fig. 3g). WT and *Gabra2*-1 spend similar amounts of time with the novel (N) object during the test session (Fig. 3f), and also show similar exploration of the objects during the familiarization session (Supplementary Fig. 3a). These results suggest that *Gabra2*-1 mice have impairments in both working and episodic memory, and tend to perseverate in exploring familiar locations and objects.

In addition to ID, a number of other behavioral symptoms are reported as a part of the human *ARHGEF9* mutation syndrome including hyperactivity, anxiety, and autism-like features. *Gabra2*-1 mice show persistently elevated levels of exploration across repeated exposures to the open field (Fig. 3b), and also across multiple phases of the novel object recognition task (Fig. 3e). To examine this hyperlocomotion further, we also monitored activity in individually housed WT and *Gabra2*-1 mice by placing beam break frames around their home cage (Fig. 3c). Cumulative activity in the home cage over a 24-hour period is dramatically increased in *Gabra2*-1 mice compared to WT littermate controls (Fig. 3c), further confirming hyperlocomotion. Anxiety-like phenotypes were next assessed using both the light-dark box and elevated plus maze. *Gabra2*-1 mice spend less time on the light side (Fig. 3h) and less time in the open arms when compared to WT littermate controls (Fig. 3i). To investigate autism-like phenotypes we used the 3-chambered social interaction paradigm, and assessed the time spent interacting with a novel social stimulus (S) or a novel object (O). We find that WT mice have a strong preference for spending time with the novel mouse over the novel object, while *Gabra2*-1 mice spend similar amounts of time with the novel mouse and novel object (Fig. 3j). We also calculated a discrimination ratio for social interaction, which is positive for WT mice indicating their social preference, while it is neutral for *Gabra2*-1 mice (Fig. 3k). Behavioral phenotyping of *Gabra2*-1 mice revealed strong parallels with frequently reported behavioral symptoms of the human *ARHGEF9* syndrome beyond ID, further validating *Gabra2*-1 mice as a useful model to study the *ARHGEF9* mutation syndrome.

### Seizures, mortality, and baseline electroencephalographic abnormalities are observed in *Gabra2*-1 mice

**Fig. 4:**
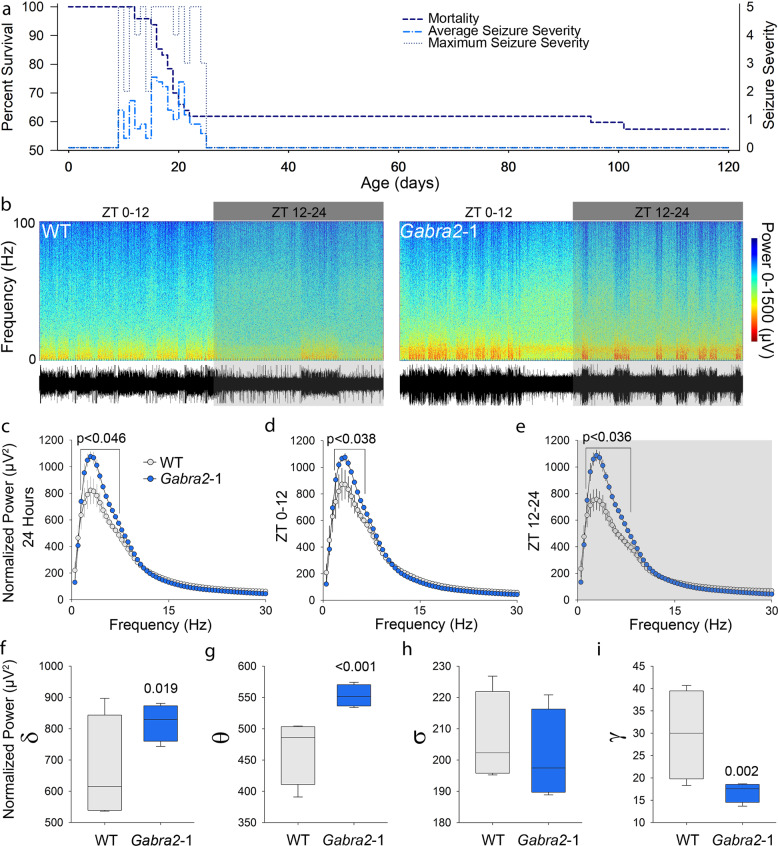
*Gabra2*-1 mice are characterized by spontaneous epilepsy during development as well as background EEG abnormalities. **a** Line plot superimposing the incidence and severity of spontaneous seizures observed in *Gabra2*-1 mice with early mortality. Mortality maps directly on to the period of time when the spontaneous seizures are observed. **b** Representative 24-hour spectrograms and traces from electroencephalogram (EEG) recordings of WT and *Gabra2*-1 mice. **c** Fast-Fourier transform analysis of complete 24-hour recordings of WT and *Gabra2*-1 mice. **d, e** Fast-Fourier transform analysis of light (ZT0-12) and dark (ZT12-24) phases of the 24 h cycle. **f** Analysis of δ waveform EEG power reveals an elevation in *Gabra2*-1 mice compared to WT controls. **g** θ waveform power is also significantly elevated in *Gabra2*-1 mice, while σ waveform power remains unchanged (**h**). **i** γ waveform power is significantly decreased in *Gabra2*-1 mice. Graphs c–e plot mean and standard error; graphs **f**–**i** plot median, first and third quartile, and range.

During our characterization of *Gabra2*-1 we observed mortality in a subset of pups during the postnatal period. To examine the mortality rate more closely we monitored the offspring from 50 litters over the course of 120 days, and calculated the percent survival of each litter (Fig. 4a). We find that mortality occurs in a specific time window between postnatal day (PND) 12 and 22, with an overall survival rate of ~62% (Fig. 4a). Retrospective genotyping revealed that all mice found dead express the *Gabra2*-1 mutation. Mortality is paralleled by the incidence of spontaneous seizures that can be observed behaviorally beginning on PND9 (Fig. 4a). Behavioral seizures vary in both average and maximum severity according to Racine scale ratings, and have not been observed after PND26 (Fig. 4a). To determine if *Gabra2*-1 mice continue to have seizures that cannot be observed behaviorally, we analyzed long-term electroencephalogram (EEG) recordings from adult mice and were unable to detect seizure activity that met any of multiple criteria applied (Supplementary Tables 5, 6). During this analysis, we did observe abnormalities in the baseline EEG, which are qualitatively evident in the low-frequency wavelengths in representative 24-hour spectrograms (Fig. 4b). To assess this quantitatively, we applied a cumulative fast Fourier transformation (cFFT) to the EEG data (Fig. 4c), revealing that *Gabra2*-1 mice have significantly increased power in low frequencies (below 10 Hz). Plots of cFFTs across the 24-hour EEG recordings show that *Gabra2*-1 mice have increased power in low frequencies irrespective of the circadian time period (Fig. 4d, e). Parsing the 24-hour data into spectral frequency bands (0.5–4 Hz–δ; 4–10 Hz–θ, 10–15 Hz– σ, 30–100 Hz–γ) reveals that *Gabra2*-1 mice have a significant increase in both δ and θ frequency band power (Fig. 4f, g), no change in σ (Fig. 4h), and a significant decrease in γ (Fig. 4i). These observations demonstrate that *Gabra2*-1 mice have spontaneous seizures during postnatal development, which resolves into persistently increased low-frequency δ and θ power across the 24-hour period, reminiscent of the documented epilepsy and baseline EEG abnormalities in *ARHGEF9* patients.

### Electroencephalographic alterations in *Gabra2*-1 mice are vigilance state specific, and associated with sleep abnormalities

**Fig. 5:**
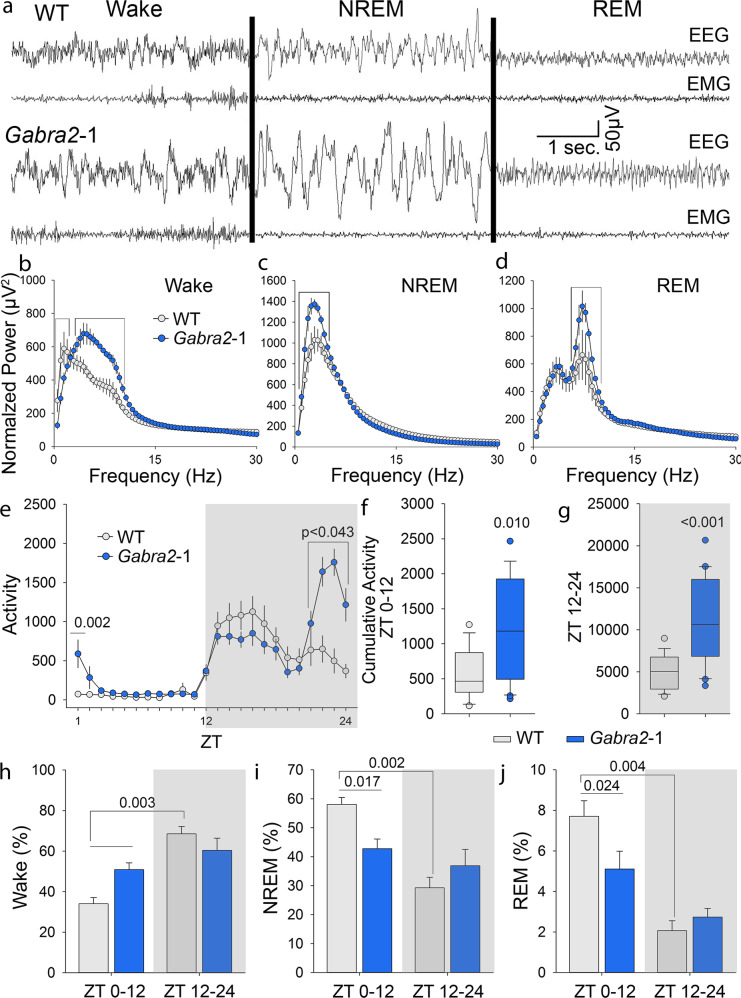
*Gabra2*-1 mice show EEG abnormalities during all vigilance states, as well as a reduction in the time spent in NREM and REM. **a** Representative EEG and EMG traces during Wake, NREM, and REM in WT and *Gabra2*-1 mice. **b–d** Fast-Fourier transform analysis of the EEG waveform during specific stages of sleep reveals an elevation in low-frequency waveform power in *Gabra2*-1 mice that is unique to each sleep stage. **e** Recording activity in the home cage reveals that *Gabra2*-1 mice are hyperactive, particularly around the transition from dark to light. **f, g**. Cumulative activity is increased in *Gabra2*-1 mice during both the light (ZT0-12) and dark (ZT12-24) phases of the cycle. **h** Epoch analysis of the EEG recordings reveals that *Gabra2*-1 mice spend significantly more time in wake during the light phase compared to WT controls, and that the time spent in wake does not differ between light and dark phases for *Gabra2*-1 mice. **i, j** *Gabra2*-1 mice spend significantly less time in NREM and REM during the light phase compared to WT controls. Graphs **b**–**e**, **h**–**j** plot mean and standard error; graphs f,g plot median, first and third quartile, and range.

Given the role of low-frequency oscillations in sleep [46], along with indications of sleep disruptions in *ARHGEF9* syndrome, we next examined vigilance states. Vigilance analysis was based on multiple consecutive 24-hour periods of EEG recording from freely moving WT and *Gabra2*-1 mice entrained to a 12-hour light-dark cycle. Qualitative visualization of raw traces of each vigilance state type (Fig. 5a) suggests that *Gabra2*-1 mice may have altered spectral characteristics, thus we plotted cFFTs of the EEG data with respect to vigilance state (Fig. 5b–d). Overall *Gabra2*-1 mice have typical power architecture in each vigilance state (broad spectrum in W, prominent δ in NREM, and prominent θ in REM), but strikingly display increased power in limited yet different frequency ranges for each vigilance state. *Gabra2*-1 mice have increased power in the δ frequency range restricted to the state of NREM, accompanied by an increase in θ and a decrease in γ power restricted to states of W and REM (Supplementary Fig. 5a–c). To examine sleep behaviorally, we individually housed diurnally entrained mice revealing periods of increased activity in *Gabra2*-1 mice that flank the transition between dark and light (Fig. 5e). Averaging across the entire 14 day period we observed an increase in the cumulative activity of *Gabra2*-1 mice during both ZT 0-12 and ZT 12-24 (Fig. 5f, g). Since activity-based assessment is limited by the fact that it cannot detect subtle vigilance states such as quiet wakefulness, we next parsed the EEG into 8 s epochs and scored as W, NREM, or REM determined by both the EEG and EMG signal characteristics. Examination of the percent time animals spent in each vigilance state again suggests sleep abnormalities in *Gabra2*-1 mice (Fig. 5h–j). *Gabra2*-1 mice spend significantly more time in W during subjective night (ZT 0-12; Fig. 5h) at the expense of time in both NREM and REM (Fig. 5i, j). We also examined circadian cycling in *Gabra2*-1 mice by exposing them to constant darkness (D/D) for 14 days. WT mice maintain a free-running circadian rhythm for the entirety of the 14 days of D/D, while *Gabra2*-1 mice display an aberrant increase in activity evident during the first introduction of darkness during ZT 0-12 (Supplementary Fig. 6a–c). Because *Gabra2*-1 mice have elevated activity under diurnal conditions, we also created a ratio of the activity between ZT 0-12 and ZT 12-24. The ratio of activity in WT mice is relatively low in L/D conditions and is not significantly altered in WT mice exposed to D/D. *Gabra2*-1 mice are more active across the entire 24-hour period producing an activity ratio comparable to WT, yet when exposed to D/D the activity ratio increases substantially in *Gabra2*-1 mice (Supplementary Fig. 6d). These experiments show that *Gabra2*-1 mice have altered EEG power during all vigilance states, but these alterations are restricted to specific frequencies relevant to each vigilance state. These alterations in vigilance state EEG characteristics manifest as increased time awake, along with impairments in circadian regulation of sleep.

We next examined biological indicators of vigilance regulation. While prominent δ power is associated with NREM sleep, high θ frequencies are associated with W and transitioning amongst these states [47, 48]. We plotted a ratio of θ to δ frequency power when binned every 30 min over a 24-hour period (Supplementary Fig. 7a). WT mice maintain a θ / δ power ratio below 1, indicating that δ power exceeds that of θ, while values above 1 are observed during specific time points and on average during ZT 12-24 in *Gabra2*-1 mice (Supplementary Fig. 7a, b), particularly around the transition between light and dark (ZT 10-14; Supplementary Fig. 7c). This analysis suggests that *Gabra2*-1 mice do not regulate the relationship among these behaviorally relevant frequency ranges in the same way as WT littermates. Extending from our findings that *Gabra2*-1 mice have a decrease in time spent asleep during subjective night, and deficits in regulation of frequencies associated with vigilance states of NREM and REM, we next examined vigilance state duration and transitions. A qualitative examination of 24-hour actograms (Supplementary Fig. 7d) suggests altered vigilance state transitions and bout duration (time between transitions) in *Gabra2*-1 mice compared to WT controls. To quantitatively assess this observation, we determined the mean bout duration of W, NREM, and REM with respect to subjective day and night. *Gabra2*-1 mice show a striking increase in mean bout duration of W during subjective night (Supplementary Fig. 7e), which is not present during subjective day (Supplementary Fig. 6F). To corroborate these data, we next plotted the total number of transitions occurring over 24-hours and find that *Gabra2*-1 mice have a decrease in vigilance state transitions (Supplementary Fig. 7g), particularly transitions into and out of REM (Supplementary Fig. 7d, e). Taken together, our findings demonstrate that *Gabra2*-1 mice have reduced time spent in sleep states, interrelated with a reduction in state transitions during sleep.

## Discussion

The human *ARHGEF9* syndrome is characterized by ID, epilepsy, and baseline EEG abnormalities, as well as a high incidence of behavioral symptoms common to other neurodevelopmental disorders including hyperactivity, anxiety, and autism-like traits. Of interest, sleep dysfunction is relatively consistently reported in patients with *ARHGEF9* mutation, and has been identified as a component of a number of other types of syndromic ID including Down syndrome [49–51]. Patients with *ARHGEF9* mutation are reported to have disrupted sleep-wake cycle [4], spike-wave discharges during slow wave sleep [5], as well as NREM parasomnias [23], and obstructive sleep apnea [6]. In some of the case reports, sleep was not necessarily excluded from the syndrome but as yet not reported on as a component of the syndrome, thus it is possible that the prevalence of sleep disturbance is greater than known to date. Additional EEG studies in human *ARHGEF9* patients are needed to understand the prevalence and characteristics of the sleep dysfunction, and also to allow comparison of the seizure characteristics, and specific features of the baseline EEG that are abnormal. This analysis also suggests that large and systematic studies [4] that employ standardized definitions and validated metrics of the clinical features [52] in *ARHGEF9* patients will be required to fully characterize the syndrome.

Given that many of the mutations in *ARHGEF9* are missense, or partial to full deletions, it is likely that human *ARHGEF9* ID syndrome results from loss of Cb function and downstream molecular consequences. In addition, mutations influencing each of Cb’s functional domains appear to result in an ID syndrome with some commonalities, suggesting that each of these domains is required for typical neurodevelopment. Prior studies have shown that the PH domain interacts with the SH3 domain to hold Cb in a closed or inactive state, and that interaction between the SH3 domain and binding partner NL2 stabilizes the active Cb conformation [26]. The Cb knockout mouse has been reported to have impaired learning, convulsions, and also anxiety [24, 25], drawing multiple parallels to human *ARHGEF9* mutation. To understand the mechanisms of dysfunction further, we have specifically probed the interaction between Cb and the GABA_A_R α2 subunit. Introducing a substitution mutation into the Cb binding sequence within the large intracellular loop of the α2 subunit (*Gabra2*-1) results in a loss of interaction with Cb and a dramatic reduction in Cb expression. In the *Gabra2*-1 mice the α2 subunit is modified with a sequence from the α1 subunit that binds gephyrin. Thus, the increase in steady state accumulation of the mutant subunit may arise at multiple levels that are independent of Cb. These include increased stability of the mutant α2-1 subunit at subtypes of synapses due to interaction with gephyrin [53], changes in the rate of oligomerization with β and γ2 subunits within the ER leading to altered retro-translocation and degradation rates [54–56], or modifications in receptor endocytosis, endocytic trafficking and lysosomal degradation [55, 57–59]. In contrast, the loss of Cb in *Gabra2*-1 mice may be related to reduced ability to bind to the large intracellular loop of the α2-1 mutant subunit and thus impaired retention at synaptic sites.

The effect of the *Gabra2*-1 mutation on Cb expression is relatively specific, as no significant change was observed for expression of gephyrin, NL2, the α1, and α3 subunits, or KCC2 and NKCC1. The *Gabra2*-1 mutation appears to influence a subset of inhibitory synapses since Cb clusters still colocalize with Syt2 and PV clusters on the soma of cortical cells in *Gabra2*-1 mice [29]. The loss of Cb cluster intensity was specific to clusters of CB1Rs and CCK on the soma of cortical cells, where reduced colocalization of Cb with these markers was also found. Cb in *Gabra2*-1 mice is still present at CCK-basket cell synapses as evidenced by positive colocalization values, thus it is likely that the *Gabra2*-1 mutation influences the stability or retention of Cb at these sites. Taken together with prior findings of a loss of inhibitory synapses on the AIS of *Gabra2*-1 mice [29], these studies suggest that interrupting the interaction of Cb and the α2 subunit has inhibitory subtype specific consequences. These findings link the GABA_A_R α2 subunit to Cb as a critical interacting partner.

The dramatic down regulation of Cb expression in the *Gabra2*-1 mouse provides construct validity, thus we next pursued phenotypic analysis to determine if the model also possesses face validity relevant to the features of the human *ARHGEF9* mutation syndrome. *Gabra2*-1 mice display phenotypes relevant to ID using multiple assays used to measure both working and recognition memory, each of which are established components of human ID [1, 43–45]. We also find phenotypes indicative of hyperactivity, anxiety, and impaired social interaction, all of which are reported with high prevalence in the *ARHGEF9* mutation syndrome. The presence of the full complement of human *ARHGEF9* behavioral symptoms in the *Gabra2*-1 mouse suggests that it may be a valuable model to understand the underlying pathophysiology, as well as provide a platform to test novel therapies.

In addition to the behavioral symptoms in *ARHGEF9* mutation syndrome, there is a high incidence of epilepsy, and also relatively frequent reports of abnormalities in the baseline EEG. Epilepsy and baseline EEG abnormalities have emerged as common hallmarks of a number of ID syndromes. Broadly, the incidence of epilepsy in ID is approximately 20%, and becomes more prevalent with increasing severity of ID [60, 61]. *Gabra2*-1 mice have spontaneous seizures during postnatal development, which can result in mortality. Seizure events are behaviorally observable in *Gabra2*-1 mice beginning on PND9, and occurring sporadically until PND26. This seizure-prone time frame overlaps with the mortality observed in *Gabra2*-1 mice between PND12 and 22. This data suggests that the mortality is related to seizure events, but not all *Gabra2*-1 mice that have seizures are subject to mortality. To determine if seizures continue into maturity despite limited mortality we examined long-term recordings from *Gabra2*-1 mice compared to WT littermate controls, and failed to find any events that met the criteria for classification as a seizure. This suggests that seizures either resolve in *Gabra2*-1 mice that survive to maturity or become exceedingly rare.

From our long-term EEG recordings, we also note abnormalities in the baseline EEG, including elevated power in low-frequency bands of δ and θ wavelength. Abnormalities in baseline EEG have also been reported in other types of ID, including Down syndrome. Baseline increases in θ band power have been noted in both mouse models [62] and patients [63] with Down syndrome. Synchronized oscillatory activity has been shown to be interrelated with cognitive performance [64, 65]. In particular, increases in synchronized activity in hippocampal θ and γ are thought to play an important role in memory formation via regulation of synaptic plasticity [66, 67]. While θ is thought of as a critical marker of active location and locomotion encoding [68, 69], γ has been proposed to play a role in information transfer and spike-timing-dependent plasticity [64, 70]. Importantly, θ and γ are tightly coupled and they occur simultaneously in both hippocampus and cortex, and further, the power and coherence of both θ and γ fluctuate with the demands of the task being performed [71, 72], suggesting the need for dynamic regulation of these interrelated frequencies. In *Gabra2*-1 mice, increased power in cortical θ during W (and REM sleep) is accompanied by decreased power in γ. These findings suggest that the relationship between cortical θ and γ frequencies has been fundamentally altered in *Gabra2*-1 mice, with implications for regulation of cognitive processes.

Interrelated with the observed EEG abnormalities, sleep abnormalities are also commonly reported in patients with *ARHGEF9* mutation. Alterations in the time spent and the characteristics of each vigilance state are noted in *Gabra2*-1 mice, along with alterations in circadian control of the sleep-wake cycle in the absence of light as a Zeitgeber. In addition to their localization to perisomatic synapses in the cortex, α2-containing receptors also have unique regional expression profiles in hypothalamic and pontine regions [73–76], including on histaminergic neurons of the tuberomammillary nucleus (TMN) [77–79] and in the suprachiasmatic nucleus (SCN) [80]. TMN histaminergic neurons are wake-promoting, and sleep can be induced and maintained by prolonging IPSCs on just histaminergic neurons of the TMN [81], thus *Gabra2*-1 mice may have subtle deficiencies in inhibition onto TMN histaminergic neurons contributing to increased time in W. GABAergic signaling is also known to be necessary for SCN function including phase shifting of the light dark cycle [82] again suggesting that subtle defects in GABAergic signaling within the SCN may play a role in the loss of the free running rhythm in *Gabra2*-1 mice. These findings parallel the human reports of disrupted sleep-wake cycle [4], and abnormal discharges during NREM [4] in patients with mutation in *ARHGEF9*. Again, sleep dysfunction is relatively widely reported in ID syndromes, with core sleep abnormalities in Down Syndrome including prolonged latency to sleep, sleep fragmentation, and reduced time spent in REM [49, 50].

This research provides an increasingly comprehensive view of the ID syndrome caused by mutation in *ARHGEF9*. The striking phenocopy of the human *ARHGEF9* ID syndrome upon mutation of the α2 subunit and disruption of Cb expression suggests that GABA_A_Rs containing the α2 subunit may be central to the dysfunction. These studies also demonstrate both the construct and face validity of the *Gabra2*-1 mouse model for mechanistic investigation of the human *ARHGEF9* mutation syndrome and provide a platform for the development and assessment of further novel therapeutic strategies to treat this complex disease. Further, these mechanisms may apply more widely to other ID syndromes that share similar characteristics to the *ARHGEF9* syndrome. In particular, the α2 subunit has also been identified to be a mechanistic contributor to the ID syndrome caused by mutation in the Non-POU domain containing octamer binding protein (NONO) [83]. Other studies have linked syndromic ID to fibroblast-growth factor 13 (FGF13) [84], which has been shown to be critical for the development of α2 enriched inhibitory synapses on the AIS [85]. Dystroglycanopathies are also associated with ID [86, 87], and dystroglycan has been identified as critical in the development of α2 enriched CCK-positive basket cell synapses [88]. Taken together these findings suggest that α2 subunit signaling may be a hub for cortical development, and central to the mechanism for multiple human ID syndromes. The α2 subunit may contribute to both initial and ongoing pathology in subtypes of ID, related to its neurotrophic role in early neurodevelopmental processes [89–92], and later in its role as a key constituent of inhibitory synapses from CCK basket cells onto the soma, and chandelier cell synapses onto the AIS [31, 32]. Of interest, epidiolex (synthetic cannabidiol) acts as a negative allosteric modulator at CB1Rs [93] to ultimately promote release of GABA from CCK basket cell terminals [94, 95], contributing to its antiepileptic activity. Thus, epidiolex may have applicability to subtypes of ID that are related to GABA_A_R α2 subunit dysfunction. In addition, benzodiazepine-like modulators have been designed to preferentially target α2 subunit containing receptors, supporting the feasibility of α2 as a mechanistic target via positive allosteric modulation of GABA_A_Rs containing this unique subunit.

## Supplementary information

Supplemental Material
