# Supplementary material for: Human ARHGEF9 intellectual disability syndrome is phenocopied by a mutation that disrupts collybistin binding to the GABAA receptor α2 subunit
Source: Mol Psychiatry. 2022 Feb 15;27(3):1729–41. doi: 10.1038/s41380-022-01468-z (PMC9095487; doi:10.1038/s41380-022-01468-z)
Supplement: Supplementary file 1 — Supplemental Material [file 41380_2022_1468_MOESM1_ESM.docx]

**Supplementary Information**

**Human *ARHGEF9* intellectual disability syndrome is phenocopied by**

**a mutation that disrupts collybistin binding to the GABA_A_ receptor α2 subunit**

**One sentence summary:** GABA_A_R α2 subunit dysfunction is a core component of syndromic intellectual disability.

Dustin J. Hines^1^, April Contreras^1^, Betsua Garcia^1^, Jeffrey S. Barker^1^, Austin J. Boren^1^, Christelle Moufawad El Achkar^2^, Stephen J. Moss^3^, Rochelle M. Hines^1*^

1. Department of Psychology, University of Nevada Las Vegas, Las Vegas, Nevada, USA
2. Department of Neurology, Boston Children's Hospital, Boston, Massachusetts, USA
3. Department of Neuroscience, Tufts University School of Medicine, Boston, Massachusetts, USA

Correspondence:

Dr. Rochelle M. Hines

Department of Psychology, Psychological and Brain Sciences, Interdisciplinary Neuroscience

University of Nevada Las Vegas

rochelle.hines@unlv.edu

**Supplementary Methods**

*Study Design*

Non-transgenic littermates were used as controls in all experiments (wildtype; WT). Germ line transmission of the transgene was detected using PCR with primers spanning the intronic region which contained the remaining loxP site [1]. For mortality and spontaneous seizure studies, as well as western blotting and confocal microscopy experiments, both male and female offspring were used. In these studies, sex was examined as a biological variable (enabled by retrospective SRY PCR to determine sex as necessary), with no effect of sex being found in the analysis. EEG and behavioral assessments were conducted on coded *Gabra2*-1 and wildtype mice in cohorts of males.

*Mouse generation and maintenance*

Animals were cared for according to the NIH Guide for the Care and Use of Laboratory Animals and protocols were approved by the Institutional Animal Care and Use Committee (IACUC) of Tufts University School of Medicine or the IACUC of the University of Nevada Las Vegas. Animals were maintained at the vivarium for Tufts University’s Boston campus, as well as at the University of Nevada Las Vegas vivarium in temperature- and humidity-controlled facilities. Mice were group housed in individually ventilated cages with cob bedding and additional nesting material, with a 12-hour light-dark cycle (with the exception of the constant darkness experiments described below). Subjective night (lights on) is defined as zeitgeber time (ZT) 0-12 and subjective day (lights off) as ZT 12-24.

*Western Blotting and Immunohistochemistry*

For western blotting, brain tissue samples (n=6 mice per genotype in 2 independent experiments) were rapidly harvested and homogenized in TEEN buffer (50 mM Tris-HCL, 1 mM EGTA, 150 mM NaCl) supplemented with a protease inhibitor mixture (Roche Applied Science). Following homogenization, the protein concentration was assessed using a BCA assay (Pierce) and heated to 65ᵒC in SDS page sample buffer with 10% β-mercaptoethanol for 15 min. After SDS page, immunoblot signals (antibody details are provided in Supplementary Table 7) were detected using fluorescent secondary antibodies on a LI-COR Odyssey. For quantification, specific protein levels were determined by densitometry and were normalized to actin loading densitometry results. Results were quantified using FIJI ImageJ.

For histology and immunohistochemistry (n=6 mice per genotype in two independent experiments) animals were transcardially perfused with a periodate lysine paraformaldehyde fixative solution. Tissue sections were cut on a cryostat at thicknesses of 40 µm and were incubated in blocking solution (2.5% Bovine Serum Albumin, 5% Normal Goat Serum, 0.1% triton-x, 0.02% sodium azide in PBS) for 45 minutes, followed by primary antibody (antibody details are provided in Supplementary Table 7) incubations diluted in modified blocking solution (2% Normal Goat Serum) overnight at 4°C, and secondary incubations diluted in modified blocking solution for 1-hour at room temperature. Immunostaining intensity, density and colocalization was quantified using FIJI ImageJ.

*Behavioral analysis*

*SHIRPA and Open Field*

We conducted a basic phenotype screen using the modified SHIRPA protocol established by the European Mouse Phenotyping Resource of Standardized Screens (EMPReSS), designed to evaluate the basic phenotype of transgenic mouse strains. All modified SHIRPA assessments performed on *Gabra2*-1 mice retuned normal results. The Open Field was also based on the SHIRPA specifications with mice placed individually into the 44 x 44 cm open field arena and allowed to freely explore for 30 minutes per session. Mice were exposed to the open field between ZT 17-20, over three consecutive days. Recordings taken from above were digitized and analyzed using AnyMaze software.

*Spontaneous Alternation*

Spontaneous alternation was tested in a T-maze and began with placing animals in the start arm of the maze for free exploration following the protocol by Deacon et al [2, 3]. Once mice made a choice between the two goal arms of the T, a partition was lowered to keep the mouse within that arm. After 30 seconds the mouse was retrieved, the partition was raised and the mouse was again placed in the start arm to explore freely. If the mouse chose to explore the previously unexplored arm, the trail was scored as correct. Mice were given three such trials per day, over the course of two days. The overall score was calculated as percent correct.

*Novel Object Recognition*

The novel object recognition test was based on the protocol by Leger et al [4]. During the first day mice were given two 10-minute sessions of exploration separated by 6 hours to allow for habituation to the arena. On the second day mice were given a 10-minute familiarization session with 2 identical objects (pseudo-randomized between a Lego Duplo block stack or a plastic lab flask). 6 hours later mice were given a test session consisting of one familiar object and one novel object. Recordings taken from above were digitized and analyzed by a blinded observer to examine object exploration time. Criteria for exploration included time when the animal’s head was oriented toward the object, in close proximity (within 5cm), as well as when the animal was rearing onto the object to explore it. Time spent on top of the objects was not included as exploration.

*Anxiety*

For the light-dark exploration test, mice were placed into the light chamber of a standard 2- chamber arena [5, 6]. The arena was composed of a larger chamber that is transparent and brightly illuminated from above, and a smaller chamber that was black walled and not illuminated. The two chambers were separated by a partition with a small doorway to allow the animal to freely pass from chamber to chamber while exploring for 10 minutes. Videos were digitized and analyzed using ANYmaze to measure time spent in each chamber, as well as other parameters such as stops. The elevated plus maze was constructed based on that previously described [5] and was composed of two open and two closed arms extending from a central platform, elevated to a height of 40 cm above the floor. Test sessions were initiated by placing individual animals in the center square facing an open arm, and were terminated after 5 minutes of free exploration. Digitized video recordings (30 fps) were scored using ANYmaze for entries (all four paws into the arm) into open and closed arms, and time spent in open and closed arms.

*Social Behavior*

Social approach behavior was assessed based on a previously described protocol [6, 7]. The dimensions of each of the three chambers of the apparatus were 20cm (L) x 40.5cm (W) x 22cm (H). In the 3-chambered social behavior paradigm, test-mice were placed in the center (empty) chamber and can access chambers to the left and right, which contain small cylindrical mesh cages. After habituation to the empty apparatus (10 min), the test mouse was blocked in the center chamber while a novel mouse (FVB – 6 week old juvenile) and a novel object (small rubber ball) were introduced within the mesh cages in the left and right chambers (randomized respectively). The test-mouse was then allowed to explore the apparatus again (20 min) and was monitored for preference of the novel mouse or novel object chambers. Parameters in different zones of the three chambered apparatus were assessed using ANYmaze from digitized video recordings, by defining each of the three chambers as separate zones, and then assessing the time spent in each zone, along with other behavior parameters.

*Activity Assessment*

To analyze the activity of WT and *Gabra2*-1 mice, animals were individually housed in home cages equipped with photobeam frames (TSE Systems), housed within a sound attenuating chamber. Mice were allowed to acclimate to individual housing and the new environment for one week prior to commencement of activity monitoring for a minimum of 14 days. Beam break data was either binned with respect to circadian time (1-hour bins), or collapsed over circadian time periods (ZT 0-12; 12-24) to assess cumulative activity.

*Constant Darkness*

For constant darkness (D/D) assessment of circadian periodicity WT and *Gabra2*-1 littermates (n=6 mice per genotype in three independent experiments) mice were individually housed in home cages equipped with photobeam frames (TSE Systems), housed within a sound attenuating chamber. Mice were allowed to acclimate to individual housing and the new environment for one week prior to commencement of activity monitoring for a minimum of 14 days under normal diurnal conditions (L/D). After the minimum of 14 days of L/D, animals were exposed to D/D for 14 days. Beam break data was collapsed over circadian time periods (ZT 0-12; 12-24) to assess cumulative activity, comparing L/D to D/D.

*Electroencephalography*

Electroencephalography (EEG) and electromyography (EMG) electrodes were implanted under isoflurane anesthesia (n=4 mice per genotype in two independent experiments). For implantation of EEG electrodes, the skull surface was exposed, and four insulated wire electrodes were placed and screwed as follows: two extradural cortical electrodes were inserted bilaterally in the frontal areas and the two others were inserted bilaterally in the parietal/occipital areas. For implantation of EMG, two insulated wire electrodes were inserted bilaterally into the nuchal muscle. Following implantation animals were individually housed. Electrodes connected to a microconnector (Pinnacle Technology) were secured at the surface of the skull with dental acrylic. After a minimum of 5 d of postoperative recovery and one week of habituation to the recording chamber and tether, EEG activity was measured using the Pinnacle system for mouse for a minimum of three weeks of 24-hour recording. The recording devices were set up in a private, sound attenuated room to provide minimal disruption. EEG and EMG signals were band-pass filtered at 1–100 Hz, digitized at 200 Hz, and acquired with a computer-based system and Sirenia software (Pinnacle Technology). EEG data was subjected to fast Fourier transformation, and this data was then used to detect power difference in 0.5-4 Hz – δ; 4-10 Hz – θ, 10-15 Hz – σ, 30-100 Hz – γ frequencies. Each hour of EEG data was scored by offline visual inspection for the appearance of abnormal events by genotype blinded experimenters. Results were quantified using SleepSign and MatLAb.

*Vigilance state scoring and analysis*

Vigilance states were scored visually based on 4 s epochs by a trained experimenter using SleepSign for Animal software (Kissei Comtec). Wakefulness (W) consisted of low amplitude, high frequency EEG and high EMG activity; rapid eye movement (REM) sleep consisted of low amplitude, desynchronized EEG with low EMG activity; and non-rapid eye movement (NREM) sleep consisted of high-amplitude, low frequency EEG with little EMG modulation. Brief awakenings defined as uninterrupted waking episodes of 1–4 s epochs were not included in the analysis. Epochs containing movement artifacts were included in the state totals. After assignments of state scores, the amount of each state (expressed as a percentage of the total recording time in 1-hour time bins) and their duration were measured. The data from each scored vigilance state was then transformed using a fast Fourier transformation and normalized to total power of individual animals. The resulting data was also parsed into biologically relevant frequency rages (0.5-4 Hz – δ; 4-10 Hz – θ, 30-100 Hz – γ). Data extracted was also used to plot the percent time spent in each vigilance state, bout duration, and the number of transitions between vigilance states. Results were quantified using SleepSign and MatLAb.

**References**

1. Hines RM, Maric HM, Hines DJ, Modgil A, Panzanelli P, Nakamura Y, et al. Developmental seizures and mortality result from reducing GABAA receptor α2-subunit interaction with collybistin. Nat Commun. 2018;9.

2. Deacon RMJ, Rawlins JNP. T-maze alternation in the rodent. Nat Protoc. 2006;1:7–12.

3. Hines RM, Hines DJ, Houston CM, Mukherjee J, Haydon PG, Tretter V, et al. Disrupting the clustering of GABAA receptor α2 subunits in the frontal cortex leads to reduced γ-power and cognitive deficits. Proc Natl Acad Sci U S A. 2013;110:16628–16633.

4. Leger M, Quiedeville A, Bouet V, Haelewyn B, Boulouard M, Schumann-Bard P, et al. Object recognition test in mice. Nat Protoc. 2013;8:2531–2537.

5. Holmes A, Kinney JW, Wrenn CC, Li Q, Yang RJ, Ma L, et al. Galanin GAL-R1 Receptor Null Mutant Mice Display Increased Anxiety-Like Behavior Specific to the Elevated Plus-Maze. Neuropsychopharmacology. 2003;28:1031–1044.

6. Hines RM, Wu L, Hines DJ, Steenland H, Mansour S, Dahlhaus R, et al. Synaptic imbalance, stereotypies, and impaired social interactions in mice with altered neuroligin 2 expression. J Neurosci Off J Soc Neurosci. 2008;28:6055–6067.

7. Moy SS, Nadler JJ, Perez A, Barbaro RP, Johns JM, Magnuson TR, et al. Sociability and preference for social novelty in five inbred strains: an approach to assess autistic-like behavior in mice. Genes Brain Behav. 2004;3:287–302.

**Supplementary Table 1.** Seizure characteristics of the patients characterized in the present study.

|  | **Patient 1 W65X** | **Patient 2 E429Kfs** |
| --- | --- | --- |
| Age of Onset | 20 months | 4 months |
| Seizure Type | Generalized tonic-clonic | Generalized tonic-clonic  Focal motor (eye and head deviation to left) |
| Seizure Frequency | Range from none (age 2-4y) to multiple per day (14y) | 2-4 per month |
| Triggers | Undetermined | Fever, but most are spontaneous |
| Status Epilepticus | No | No |
| Medications or Interventions | Levetiracetam: god response 2-4y; behavioral SEs  Valproic acid: good response; behavioral SEs  Clobazam; Clonazepam; Brivaracetam; Epidiolex: partially effective  Zonisamide; Lamotrigine, Topiramate: ineffective  Ketogenic diet: partially effective  Current: Valproic acid, Clobazam, Clonazepam, Epidiolex | Levetiracetam; Valproic acid; Clobazam; Diazepam; Topiramate; Zonisamide: ineffective to partially effective  Current: Oxcarbazepine; Phenobarbital; Rufinamine; Valproic acid – improved seizure frequency, but seizures are not controlled |
| EEG Findings | Ages 2; 4; 5; 13: Normal EEG; seizures were never captured on EEG | Age 3: several seizures; multifocal onset and multifocal spikes; absence of posterior dominant rhythm  Ages 12-14: mostly unchanged but reduction in spikes; intermittent generalized slowing; slow posterior dominant rhythm for age |

**Supplementary Table 2.** Major phenotypes reported in association with specific mutations in *ARHGEF9*.

| **Mutation** | **Phenotype** | **Sex** | **#** |
| --- | --- | --- | --- |
| **New Cases** | | | |
| W65X | Severe ID, Epilepsy, anxiety, Autistic features | M | 1 |
| E429Kfs | Severe ID, Epilepsy, hyperactivity, Autistic features, abnormal EEG | F | 1 |
| **Point Mutations** | | | |
| Q2X | Severe ID, Epilepsy, abnormal EEG | M | 1 |
| G55A | Severe ID, hyperekplexia, Epilepsy | M | 1 |
| R104Q | Severe ID, hyperactivity, Autistic features, Epilepsy, abnormal EEG | M | 2 |
|  | Severe ID, Epilepsy, abnormal EEG | F |  |
| L177P | Severe ID, Autistic features, Epilepsy, abnormal EEG, Sleep abnormalities | M | 1 |
| E179K | Moderate ID, Autistic features, Sleep abnormalities | F | 1 |
| R290C | Moderate to severe ID (3/4 severe, 1/4 mod), Epilepsy | M | 4 |
| R290H | Moderate ID, Epilepsy, abnormal EEG | M | 1 |
| I294T | Mild ID, Epilepsy | M | 1 |
| S317W | Severe ID, Autistic features, Epilepsy, abnormal EEG | M | 2 |
| G323R | Epilepsy, Moderate to Severe ID, hyperactivity | M | 4 |
| R338W | Moderate ID, Epilepsy | M | 1 |
| R356Q | Mild ID | M | 1 |
| R357I | Moderate ID, Epilepsy (developmental), hyperactivity | M | 1 |
| E400K | Moderate ID, Sleep abnormalities | M | 1 |
| F492L | ID (unclassified), Epilepsy | M | 1 |
| E496K | Mild ID, Sleep abnormalities (NREM parasomnias), Epilepsy | M | 1 |
| **Splice Variants** | | | |
| SV  R381+3A>G | DD, Epilepsy, abnormal EEG | M | 1 |
| SV; Exon skipping  1300+2T>C | Moderate ID | M | 1 |
| **Balanced Translocations** | | | |
| BT  46,XX,t(X;20)(q12;P13) | Severe ID, hyperactivity, Autistic features, Epilepsy, abnormal EEG | F | 1 |
| BT  46,X,t(X;18)(q11.1;q11.21) | Severe ID, hyperactivity, Epilepsy, abnormal EEG, Sleep abnormalities | F | 1 |
| **Paracentric Inversions** | | | |
| PI 46,X,inv(X)(q11.1q27.3) | Moderate ID, hyperarousal | F | 1 |
| **Deletions** | | | |
| del 7.5kb  62,854,862-62,862,403 | Moderate ID, Epilepsy | F | 1 |
| del 26.9kb at Xq11.1 | Mild DD, autistic features | F | 1 |
| del 27kb  62,838,630-62,865,334 | Moderate ID, hyperactivity, Sleep abnormalities | F | 1 |
| del 56.5kb at Xq11.1 | Mild DD, autistic features | F | 1 |
| del 82kb at Xq11.1-11.2  62,970,571e63,052,696 | Hyperactivity, Mild ID, DD, autistic features | F | 1 |
| del 216.7kb (at Xq11.1) | Severe ID, epilepsy, autistic features, abnormal EEG | M | 1 |
| del 737kb  62,321,746-63,058,549 | Severe ID, Epilepsy, abnormal EEG | M | 1 |
| del 1.29Mb  61,848,414-63,138,698 | Severe ID, hyperactivity, Epilepsy | M | 1 |
| Partial gene deletion | Severe ID, Hyperactivity | F | 1 |
| Full gene deletion | Severe ID, Epilepsy | F | 1 |

**Supplementary Table 3.** Human *ARHGEF9* mutations organized according to reported phenotype.

|  | *ARHGEF9* Mutations or Disruptions | % of Patients |
| --- | --- | --- |
| Developmental delay / Intellectual disability* | Mi: I294T; R356Q; E496K; Xq11.1-11.2 microdel; del 26.9kb at Xq11.1; del 56.5kb at Xq11.1 **(total: 6)**  Mo: E179K; R290C; R290H; R338W; R357I; E400K; [SV  R381+3A>G]; [SV; Exon skipping 1300+2T>C]; [PI 46,X,inv(X)(q11.1q27.3)]; del 7.5kb; del 27kb; G323R **(total: 12)**  S: Q2X; G55A; R104Q; L177P; S317W; R290C; [BT 46,XX,t(X;20)(q12;P13)]; [BT 46,X,t(X;18)(q11.1;q11.21)]; del 737kb; del 1.29Mb; del 216.7kb at Xq11.1; full gene deletion; partial gene deletion; G323R **(total: 14)** | Mi: 18.75  Mo: 37.50  S: 43.75  Any: 100% |
| Seizures or Epilepsy | Q2X; G55A; R104Q; L177P; R290C; R290H; I294T; S317W; R338W; R357I; E496K; SV R381+3A>G; BT 46,XX,t(X;20)(q12;P13); BT 46,X,t(X;18)(q11.1;q11.21); del 7.5kb; del 737kb; del 1.29Mb; del 216.7kb (at Xq11.1); G323R; full gene deletion **(total: 20)** | 62.50 |
| Baseline EEG abnormalities | Q2X; R104Q; L177P; R290H; S317W; SV R381+3A>G; BT 46,XX,t(X;20)(q12;P13); BT 46,X,t(X;18)(q11.1;q11.21); del 737kb; del 216.7kb (at Xq11.1) **(total: 10)** | 31.25 |
| Hyperactivity,  Hyperarousal or Anxiety | R104Q; R357I; BT 46,XX,t(X;20)(q12;P13); BT 46,X,t(X;18)(q11.1;q11.21); PI 46,X,inv(X)(q11.1q27.3); del 27kb; del 1.29Mb; G323R; partial gene deletion; del 82kb **(total: 10)** | 31.25 |
| Autism-like features | R104Q; L177P; E179K; S317W; BT 46,XX,t(X;20)(q12;P13); del 216.7kb; del 82kb; del 26.9kb; del 56.5kb **(total: 9)** | 28.13 |
| Sleep abnormalities | L177P; E179K; E400K; E496K; BT 46,X,t(X;18)(q11.1;q11.21); del 27kb **(total: 6)** | 18.75 |
| Hyperekplexia | G55A | 3.13 |

**Supplementary Table 4.** *ARHGEF9* mutation by type and genetic sex.

|  | Males | Females | Total |
| --- | --- | --- | --- |
| Microdeletions (10/31 = 32.25% of mutations) | 3 | 7 | 10 |
| Missense Mutations (15/31 = 48.4% of mutations) | 21 | 2 | 23 |
| Nonsense Mutations (1/31 = 3.2% of mutations) | 1 | 0 | 1 |
| Others (BT, SV, and PI) (5/31 = 16.1%) | 2 | 3 | 5 |
| Total (100%) | 26 | 12 | 38 |

**
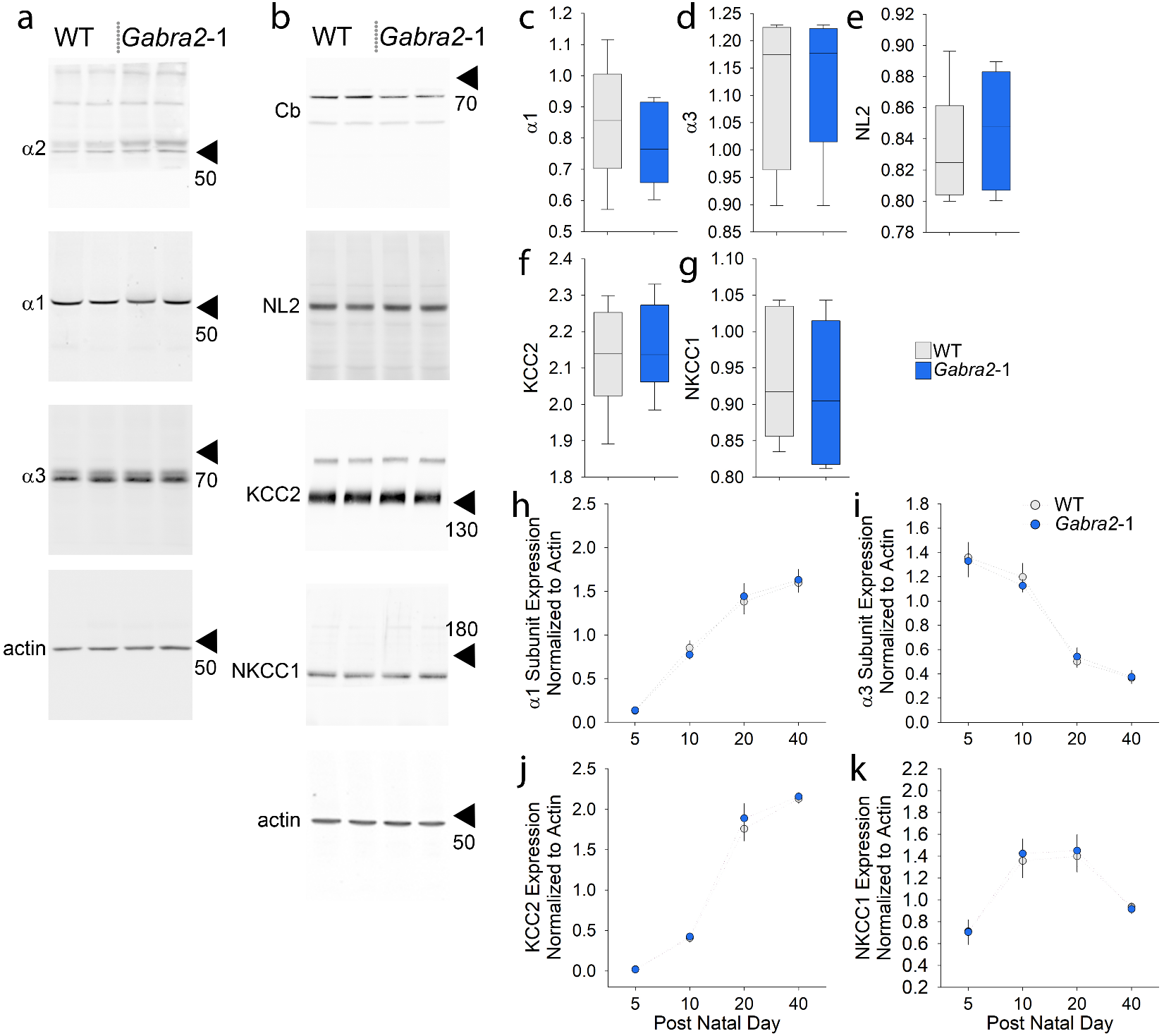
**

**Supplementary Figure 1. Assessment of protein expression changes over a developmental time course in *Gabra2*-1 mice.** a. Raw representative western blots from samples taken on PND 10 corresponding to those shown in Figure 2. b. Raw representative western blots from samples taken on PND 40 corresponding to those shown in Figure 2. c,d. Quantification of α1 (c) and α3 (d) expression at PND10 comparing cortical lysates from WT and *Gabra2*-1. e-g. Quantification of NL2 (e), KCC2 (f), and NKCC1 (g) expression at PND10 comparing cortical lysates from WT and *Gabra2*-1. h-k. Time course analysis of protein expression through postnatal development comparing cortical lysates from WT and *Gabra2*-1.

**
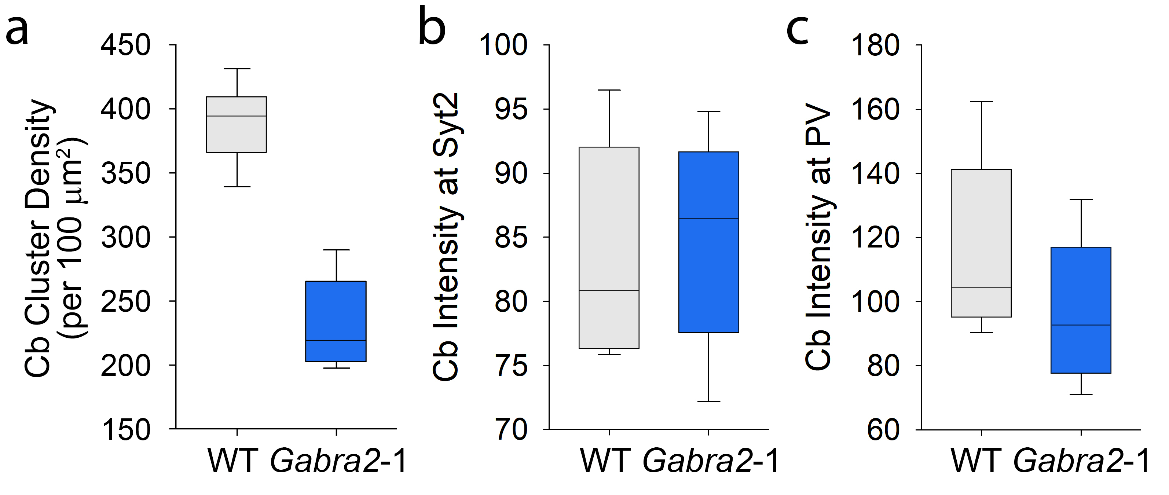
**

**Supplementary Figure 2. Collybistin cluster density and intensity.** a. Density of Cb clusters in cortical tissue comparing WT to *Gabra2*-1 mice. b. Intensity of Cb staining at sites positive for Syt2 on the soma of cortical cells in WT and *Gabra2*-1 mice. c. Intensity of Cb staining at sites positive for PV on the soma of cortical cells in WT and *Gabra2*-1 mice. Graphs plot median, first and third quartile, and range.

**
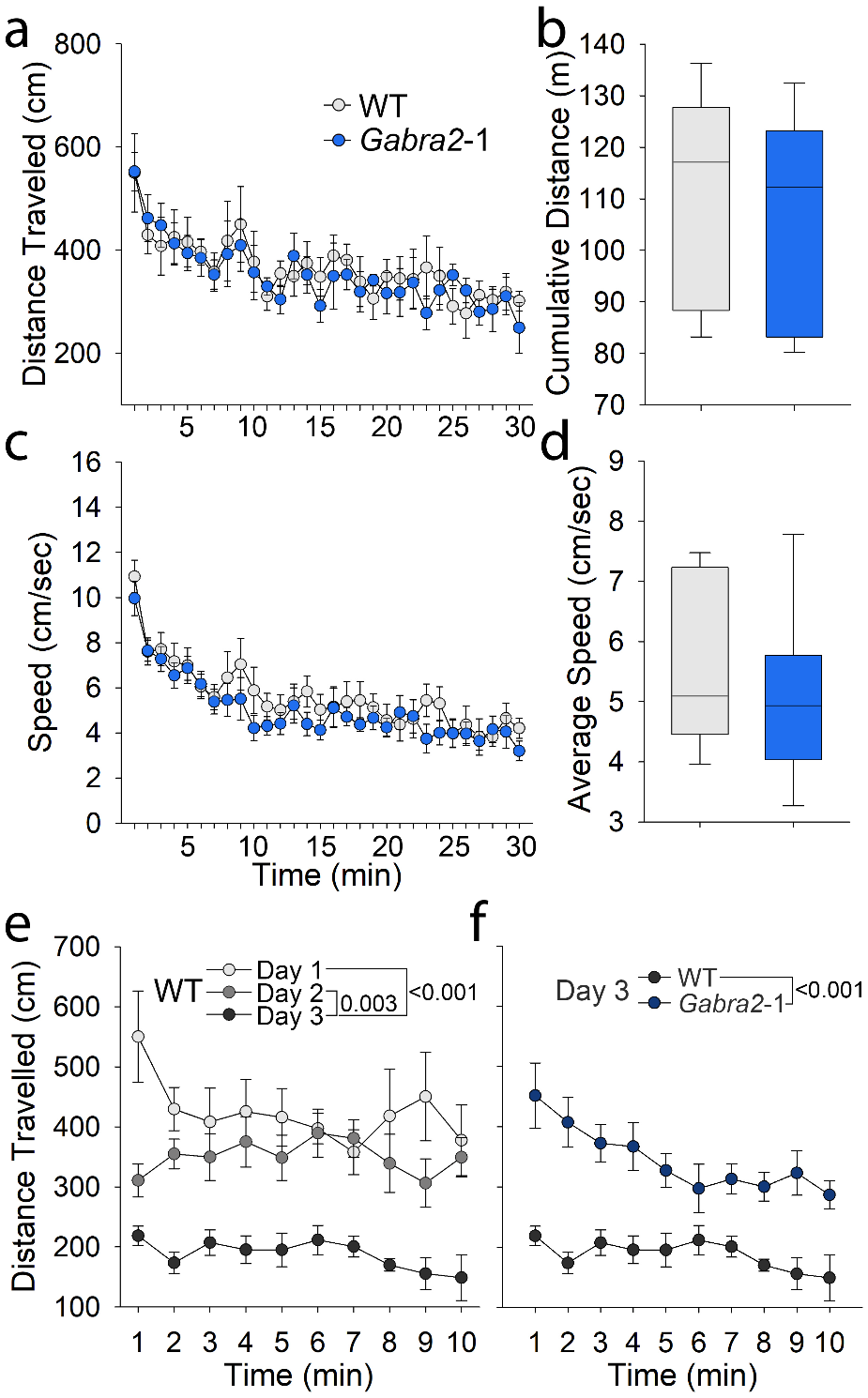
**

**Supplementary Figure 3.** **Normal open field behavior is indicative of adequate motor performance, but *Gabra2*-1 mice maintain heightened exploration over repeated exposures to the open field.** a. Distance traveled over time in the open field shows normal exploration and within session habituation in Gabra2-1 mice. b. WT and Gabra2-1 mice do not differ in cumulative distance travelled. c. Average speed of travel over time in the open field is also comparable between WT and Gabra2-1 mice. d. Average speed does not differ between WT and Gabra2-1 mice. e. Repeated exposure of WT mice to the open field on 3 successive days shows the typical inter session habituation. f. Comparison of day 3 distance travelled shows that Gabra2-1 mice maintain high levels of activity in the open field despite repeated exposure. Graphs A,C,E,F plot mean and standard error; graphs B,D plot median, first and third quartile, and range.


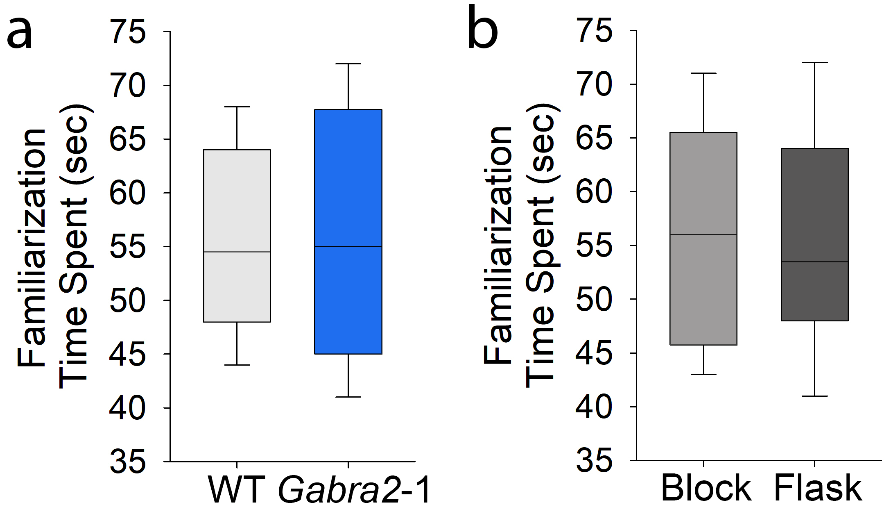


**Supplementary Figure 4.** **Object exploration during the familiarization stage is consistent across WT and Gabra2-1 mice, and also across the objects used.** a. Both WT and Gabra2-1 mice spend a similar amount of time exploring the novel objects during the familiarization stage of the novel object recognition test. b. Both the toy blocks and the flask were explored for similar amounts of time during the familiarization stage, suggesting that both have comparable salience. Graphs plot median, first and third quartile, and range.

**
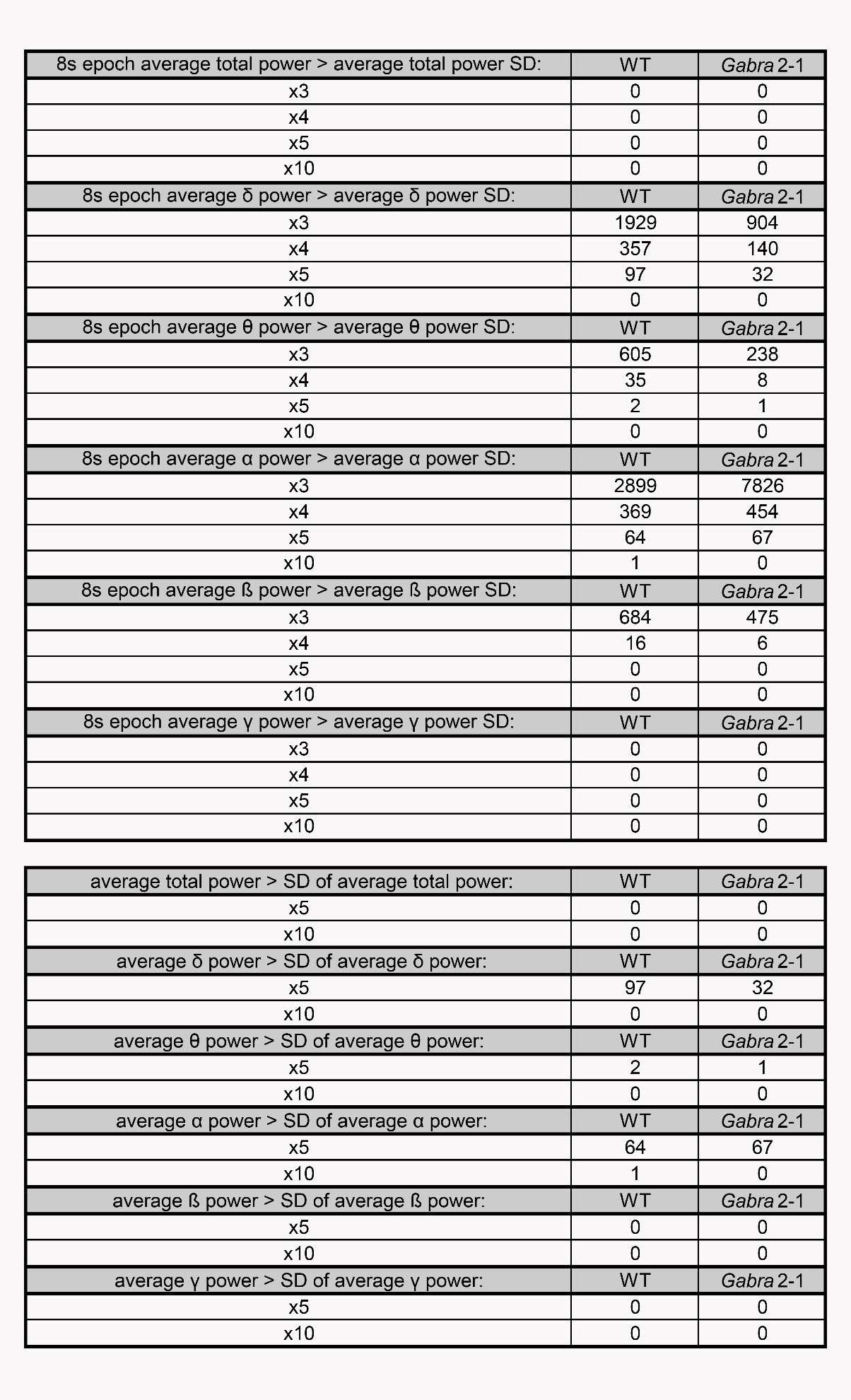
Supplementary Tables 5&6**. Seizure detection analysis of multi day EEG/EMG recordings.


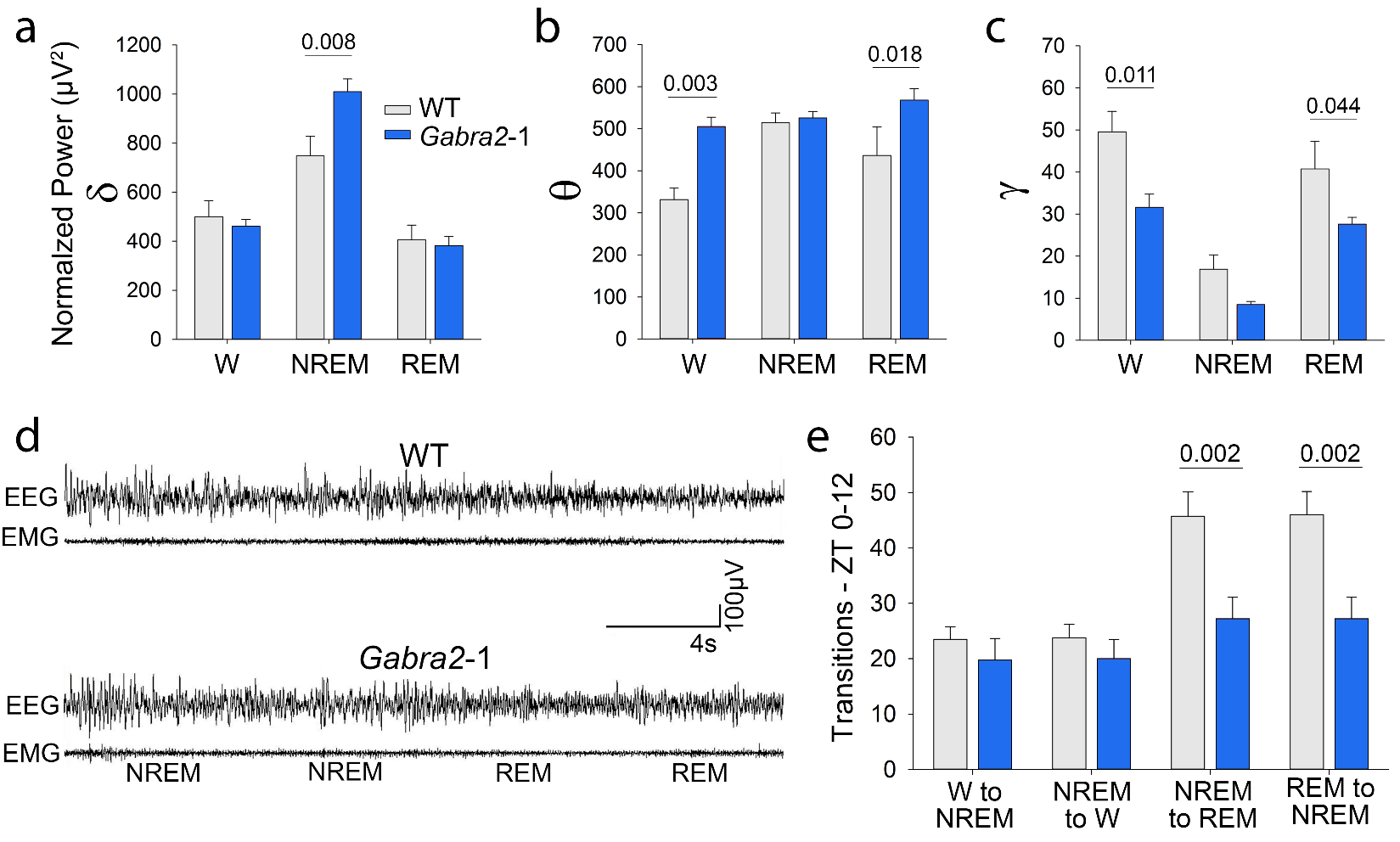


**Supplementary Figure 5.** **Detailed spectral analysis of the characteristics of sleep stages, and the transitions between stages in *Gabra2*-1 mice.** a. Spectral analysis of vigilance states reveals an increase in δ during NREM in *Gabra2*-1 mice. b. θ power is increased in both W and REM in *Gabra2*-1 mice compared to WT control. c. In contrast to the lower frequencies, γ power is suppressed in W and REM in recordings from *Gabra2*-1 mice. d. Representative EEG and EMG traces of two epochs of NREM transitioning to two epochs of REM in WT and *Gabra2*-1 recordings. e. Analysis of transitions during ZT 0-12 by type, comparing WT to *Gabra2*-1. Graphs plot mean and standard error.


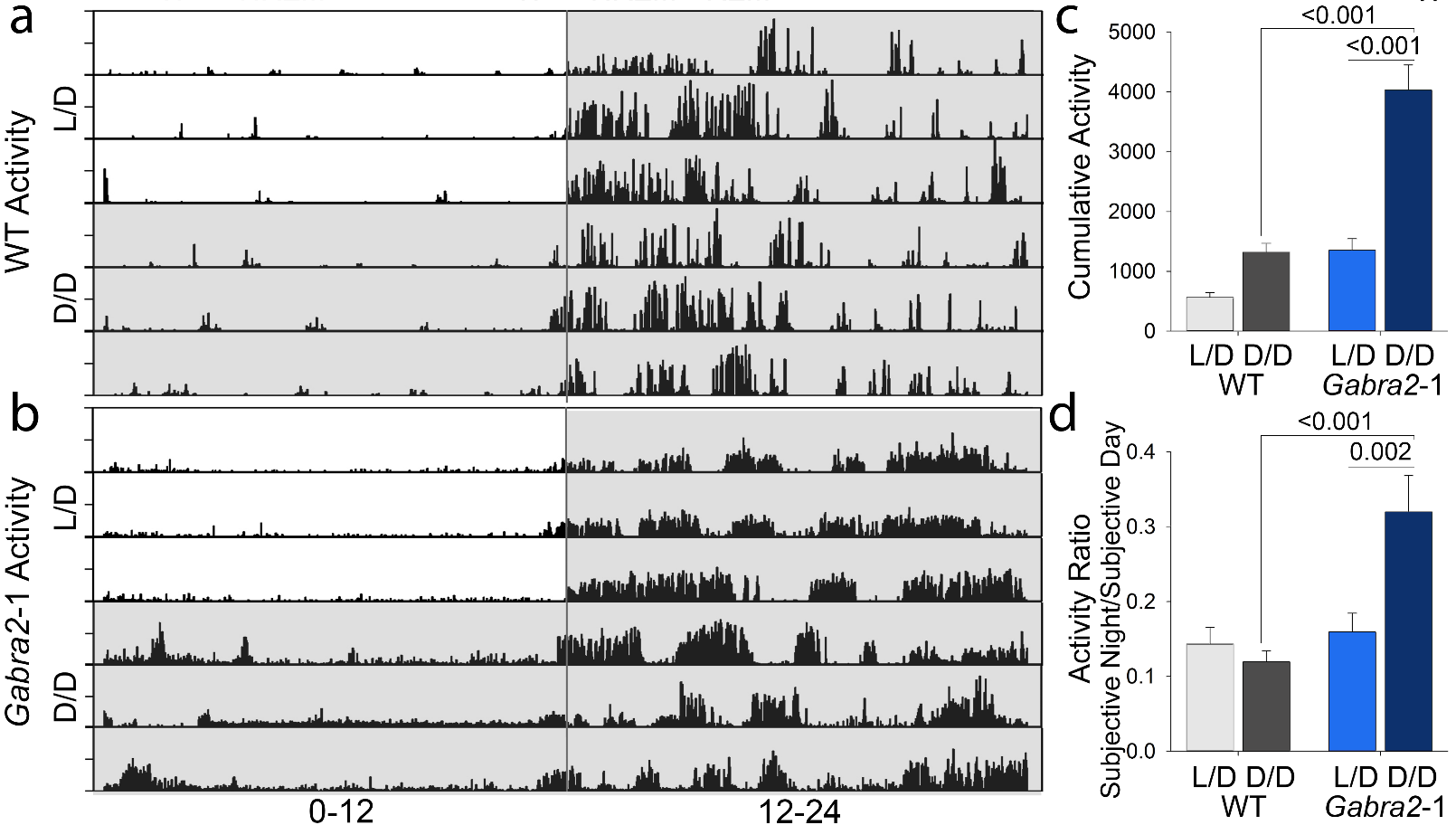


**Supplementary Figure 6.** **Abnormalities in the circadian cycle of *Gabra2*-1 mice.** a,b. Representative actograms from WT and *Gabra2*-1 mice during regular diurnal housing (light / dark – L/D) and constant darkness (dark / dark – D/D). c. Quantification of 24-hour cumulative activity in WT and *Gabra2*-1 mice, comparing L/D and D/D housing conditions. d. Activity ratio calculated by dividing subjective night (ZT 0-12) cumulative activity by subjective day (ZT 12-24) cumulative activity in WT and *Gabra2*-1 mice, comparing L/D and D/D housing conditions. Graphs plot mean and standard error.


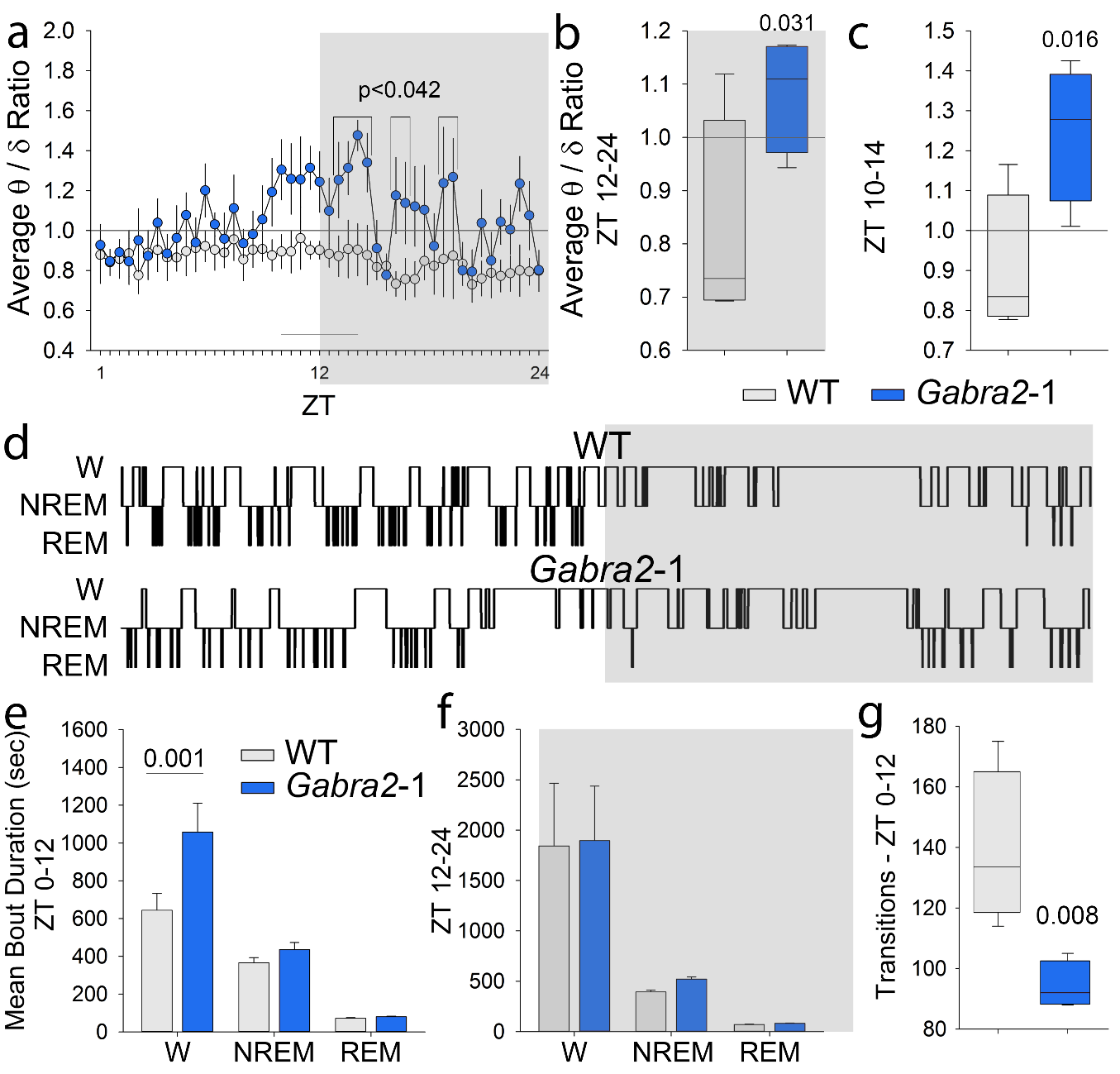


**Supplementary Figure 7. The EEG characteristics of sleep stages and the transitions between sleep stages are altered in *Gabra2*-1 mice.** a. Comparison of the average θ to δ ratio across the 24-hour cycle in WT and *Gabra2*-1 mice. b. Comparison of the average θ to δ ratio during the dark (ZT12-24) phase shows a significant elevation in *Gabra2*-1 mice. c. Examination of the average θ to δ ratio during the time surrounding the transition from light to dark reveals a strong increase in *Gabra2*-1 mice further revealing that the EEG characteristics of the sleep stages are altered. d. Representative 24-hour hypnograms of WT and *Gabra2*-1 mice. e,f. Quantification of the average bout duration for each of the sleep stages, showing that *Gabra2*-1 mice have longer bouts of wake during the light phase. g. Quantification of the total number of transitions between sleep stages reveals a significant decrease in *Gabra2*-1 mice. Graphs a,e,f plot mean and standard error; graphs b,c,g plot median, first and third quartile, and range.

**Supplementary Table 6.** Summary of Statistics.

| **Figure** | **Panel** | **n**  **(per group)** | **Test** | **Mean ± St Dev**  **OR LS Mean ± St Err LS Mean** | **df** | **Main Effects**  **P** |
| --- | --- | --- | --- | --- | --- | --- |
| 2 | d | ≥6 mice | Two Way ANOVA  Bonferroni | WT: 0.428 ± 0.0301  *Gabra2*-1: 0.701 ± 0.0308 | 48 | genotype <0.001  postnatal day <0.001 |
|  | e | 6 mice | One Way ANOVA  Bonferroni | WT: 0.587 ± 0.0291  *Gabra2*-1: 0.780 ± 0.0452 | 11 | genotype = 0.005 |
|  | f | ≥6 mice | Two Way ANOVA  Bonferroni | WT: 0.120 ± 0.00832  *Gabra2*-1: 0.0579 ± 0.0101 | 48 | genotype <0.001  postnatal day = 0.012 |
|  | g | 6 mice | One Way ANOVA  Bonferroni | WT: 0.857 ± 0.1420  *Gabra2*-1: 0.445 ± 0.0953 | 11 | genotype = 0.037 |
|  | l | 5 mice | Two-tailed t-test | WT: 0.496 ± 0.0464  *Gabra2*-1: 0.423 ± 0.0350 | 8 | ns |
|  | m | 5 mice | Two-tailed t-test | WT: 0.552 ± 0.0233  *Gabra2*-1: 0.501 ± 0.0277 | 8 | ns |
|  | n | 5 mice | Two-tailed t-test | WT: 0.256 ± 0.0489  *Gabra2*-1: 0.0956 ± 0.0350 | 8 | genotype = 0.017 |
|  | o | 5 mice | Two-tailed t-test | WT: 0.528 ± 0.0275  *Gabra2*-1: 0.193 ± 0.0414 | 8 | genotype <0.001 |
|  | p | 5 mice | Two-tailed t-test | WT: 58.731 ± 2.748  *Gabra2*-1: 46.630 ± 1.683 | 8 | genotype = 0.006 |
|  | q | 5 mice | Two-tailed t-test | WT: 76.738 ± 4.646  *Gabra2*-1: 55.470 ± 4.559 | 8 | genotype = 0.011 |
| 3 | a | 7 WT  8 *Gabra2*-1 | Kruskal-Wallis One Way ANOVA | WT: 109.457 ± 8.328  *Gabra2*-1: 105.750 ± 7.177 | 16 | ns |
|  | b | 7 WT  8 *Gabra2*-1 | Two Way RM ANOVA  Bonferroni | WT: 3203.103 ± 199.524  Gabra2-1: 4009.644 ± 186.638 | 44 | genotype x day = 0.007  genotype = 0.011 |
|  | c | 12 mice | One Way ANOVA  Bonferroni | WT: 5305.733 ± 559.684  *Gabra2*-1: 12719.632 ± 1141.791 | 22 | genotype <0.001 |
|  | d | 9 mice | Kruskal-Wallis One Way ANOVA Tukey | WT: 87.037 ± 3.704  Gabra2-1: 38.889 ± 6.804 | 15 | genotype = 0.001 |
|  | f | 9 mice | Two Way RM ANOVA  Bonferroni | WT: 31.778 ± 4.109  Gabra2-1: 46.111 ± 4.109 | 35 | genotype x object <0.001 |
|  | g | 9 mice | One Way ANOVA  Bonferroni | WT: 0.308 ± 0.0556  *Gabra2*-1: -0.147 ± 0.0372 | 15 | genotype <0.001 |
|  | h | 11 WT  7 *Gabra2*-1 | One Way ANOVA  Bonferroni | WT: 35.979 ± 3.468  *Gabra2*-1: 19.524 ± 3.538 | 17 | genotype = 0.006 |
|  | i | 5 WT  8 *Gabra2*-1 | Kruskal-Wallis One Way ANOVA Tukey | WT: 25.224 ± 7.299  Gabra2-1: 8.273 ± 2.190 | 12 | genotype = 0.019 |
|  | j | 9 mice | Two Way RM ANOVA  Bonferroni | WT: 508.333 ± 15.229  Gabra2-1: 483.133 ± 15.229 |  | genotype x target <0.001 |
|  | k | 9 mice | Kruskal-Wallis One Way ANOVA Tukey | WT: 0.260 ± 0.012  Gabra2-1: 0.020 ± 0.023 | 15 | genotype <0.001 |
| 4 | c | 4 mice | Two Way RM ANOVA  Bonferroni | WT: 258.869 ± 8.365  *Gabra2*-1: 287.789 ± 8.365 | 6 | genotype x frequency <0.001 |
|  | d | 4 mice | Two Way RM ANOVA  Bonferroni | WT: 269.787 ± 8.425  *Gabra2*-1: 289.630 ± 8.425 | 6 | genotype x frequency <0.001 |
|  | e | 4 mice | Two Way RM ANOVA  Bonferroni | WT: 245.618 ± 7.901  *Gabra2*-1: 288.289 ± 7.901 | 6 | genotype x frequency <0.001 |
|  | f | 4 mice | One Way ANOVA  Bonferroni | WT: 665.966 ± 155.245  *Gabra2*-1: 821.026 ± 54.961 | 6 | genotype = 0.019 |
|  | g | 4 mice | One Way ANOVA  Bonferroni | WT: 466.713 ± 48.690  *Gabra2*-1: 552.884 ± 16.284 | 6 | genotype <0.001 |
|  | h | 4 mice | One Way ANOVA  Bonferroni | WT: 206.669 ± 13.334  *Gabra2*-1: 201.147 ± 13.302 | 6 | genotype = 0.421 |
|  | i | 4 mice | One Way ANOVA  Bonferroni | WT: 29.746 ± 9.531  *Gabra2*-1: 16.876 ± 2.068 | 6 | genotype = 0.002 |
| 5 | b | 4 mice | Two Way RM ANOVA  Bonferroni | WT: 216.922 ± 9.261  *Gabra2*-1: 258.451 ± 9.261 | 6 | genotype x frequency <0.001 |
|  | c | 4 mice | Two Way RM ANOVA  Bonferroni | WT: 292.011 ± 5.638  *Gabra2*-1:311.061 ± 5.638 | 6 | genotype x frequency <0.001 |
|  | d | 4 mice | Two Way RM ANOVA  Bonferroni | WT: 237.574 ± 11.020  *Gabra2*-1: 267.708 ± 11.020 | 6 | genotype x frequency <0.001 |
|  | e | 12 mice | Two Way RM ANOVA  Bonferroni | WT: 422.443 ± 58.553  *Gabra2*-1: 537.708 ± 52.371 | 22 | genotype x time <0.001 |
|  | f | 12 mice | Two-tailed t-test | WT: 573.27 ± 366.40  *Gabra2*-1: 1146.33 ± 754.50 | 22 | genotype = 0.010 |
|  | g | 12 mice | Two-tailed t-test | WT: 4732.47 ± 2005.25  *Gabra2*-1: 10614.10 ± 5409.49 | 22 | genotype <0.001 |
|  | h | 4 mice | Two Way RM ANOVA  Bonferroni | WT: 51.280 ± 2.063  *Gabra2*-1: 55.622 ± 2.063 | 6 | ZT = 0.005 |
|  | i | 4 mice | Two Way RM ANOVA  Bonferroni | WT: 43.653 ± 2.790  *Gabra2*-1: 39.830 ± 2.790 | 6 | genotype x ZT = 0.026 |
|  | j | 4 mice | Two Way RM ANOVA  Bonferroni | WT: 4.886 ± 0.276  *Gabra2*-1: 3.923 ± 0.276 | 6 | genotype = 0.048  ZT = 0.003 |
| S1 | c | 6 mice | One Way ANOVA  Bonferroni | WT: 0.587 ± 0.0291  *Gabra2*-1: 0.780 ± 0.0452 | 11 | genotype = 0.005 |
|  | d | 6 mice | Kruskal-Wallis One Way ANOVA | WT: 1.114 ± 0.0566  *Gabra2*-1: 1.126 ± 0.0525 | 11 | ns |
|  | e | 6 mice | One Way ANOVA  Bonferroni | WT: 0.834 ± 0.0149  *Gabra2*-1: 0.846 ± 0.0154 | 11 | ns |
|  | f | 6 mice | One Way ANOVA  Bonferroni | WT: 2.129 ± 0.0594  *Gabra2*-1: 2.155 ± 0.0499 | 11 | ns |
|  | g | 6 mice | One Way ANOVA  Bonferroni | WT: 0.935 ± 0.0378  *Gabra2*-1: 0.915 ± 0.0419 | 11 | ns |
|  | h | ≥6 mice | Two Way ANOVA  Bonferroni | WT: 0.991 ± 0.0515  *Gabra2*-1: 0.996 ± 0.0515 | 48 | genotype ns  postnatal day <0.001 |
|  | i | ≥6 mice | Two Way ANOVA  Bonferroni | WT: 0.855 ± 0.0412  *Gabra2*-1: 0.843 ± 0.0412 | 48 | genotype ns  postnatal day <0.001 |
|  | j | ≥6 mice | Two Way ANOVA  Bonferroni | WT: 1.079 ± 0.0452  *Gabra2*-1: 1.121 ± 0.0452 | 48 | genotype ns  postnatal day <0.001 |
|  | k | ≥6 mice | Two Way ANOVA  Bonferroni | WT: 1.100 ± 0.0586  *Gabra2*-1: 1.123 ± 0.0586 | 48 | genotype ns  postnatal day <0.001 |
| S2 | c | 11 mice | One Way ANOVA  Bonferroni | WT: 389.275 ± 12.567  *Gabra2*-1: 231.122 ± 14.504 |  | genotype <0.001 |
|  | b | 5 mice | Two-tailed t-test | WT: 83.504 ± 3.846  *Gabra2*-1: 84.982 ± 3.732 | 8 | ns |
|  | c | 5 mice | Two-tailed t-test | WT: 115.467 ± 12.673  *Gabra2*-1: 96.392 ± 10.216 | 8 | ns |
| S3 | a | 7 WT  8 *Gabra2*-1 | Two Way RM ANOVA  Bonferroni | WT: 245.618 ± 7.901  *Gabra2*-1: 288.289 ± 7.901 | 6 | ns |
|  | b | 7 WT  8 *Gabra2*-1 | Kruskal-Wallis One Way ANOVA | WT: 109.457 ± 8.328  *Gabra2*-1: 105.750 ± 7.177 | 16 | ns |
|  | c | 7 WT  8 *Gabra2*-1 | Two Way RM ANOVA  Bonferroni | WT: 245.618 ± 7.901  *Gabra2*-1: 288.289 ± 7.901 | 6 | ns |
|  | d | 7 WT  8 *Gabra2*-1 | One Way ANOVA  Bonferroni | WT: 5.602 ± 0.509  *Gabra2*-1: 5.031 ± 0.455 | 16 | ns |
|  | e | 7 WT mice | Two Way RM ANOVA  Bonferroni | Day 1: 422.986 ± 26.335  Day 2: 350.365 ± 26.335  Day 3: 187.580 ± 26.335 | 209 | day <0.001 |
|  | f | 7 WT  8 *Gabra2*-1 | Two Way RM ANOVA  Bonferroni | WT: 187.580 ± 15.628  *Gabra2*-1: 344.632 ± 14.619 | 149 | genotype < 0.001 |
| S4 | a | 9 mice | One Way ANOVA  Bonferroni | WT: 55.375 ± 8.484  *Gabra2*-1: 55.750 ± 11.386 | 15 | ns |
|  | b | 9 mice | One Way ANOVA  Bonferroni | Blocks: 55.875 ± 10.077  Flask: 55.250 ± 9.996 | 15 | ns |
| S5 | a | 4 mice | Two Way RM ANOVA  Bonferroni | WT: 551.211 ± 49.232  *Gabra2*-1: 617.406 ± 49.232 | 6 | genotype x state = 0.001 |
|  | b | 4 mice | Two Way RM ANOVA  Bonferroni | WT: 427.231 ± 24.715  *Gabra2*-1: 532.895 ± 24.715 | 6 | genotype = 0.023  state = 0.014 |
|  | c | 4 mice | Two Way RM ANOVA  Bonferroni | WT: 35.717 ± 3.590  *Gabra2*-1: 22.556 ± 3.590 | 6 | genotype = 0.041  state <0.001 |
|  | e | 4 mice | Two Way RM ANOVA  Bonferroni | WT: 34.75 ± 2.38  *Gabra2*-1: 23.56 ± 2.38 | 6 | genotype x transition = 0.032 |
| S6 | a | 4 mice | Two Way RM ANOVA  Bonferroni | WT: 0.853 ± 0.0743  *Gabra2*-1: 1.060 ± 0.0743 | 6 | time = 0.002 |
|  | b | 4 mice | One-tailed t-test | WT: 0.820 ± 0.202  *Gabra2*-1: 1.084 ± 0.108 | 6 | genotype = 0.031 |
|  | c | 4 mice | One-tailed t-test | WT: 0.903 ± 0.178  *Gabra2*-1: 1.248 ± 0.173 | 6 | genotype = 0.016 |
|  | e | 4 mice | Two Way RM ANOVA  Bonferroni | WT: 360.333 ± 46.577  *Gabra2*-1: 524.333 ± 46.577 | 6 | genotype x state = 0.032 |
|  | f | 4 mice | Two Way RM ANOVA  Bonferroni | WT: 769.167 ± 194.737  *Gabra2*-1: 832.583 ± 194.737 | 6 | state <0.001 |
|  | g | 4 mice | One-tailed t-test | WT: 139.00 ± 25.73  *Gabra2*-1: 94.25 ± 7.81 | 6 | genotype = 0.008 |
| S7 | c | 12 mice | Two Way RM ANOVA  Bonferroni | WT:939.333 ± 213.531  *Gabra2*-1: 2691.700 ± 190.988 | 22 | genotype x LD/DD <0.001 |
|  | d | 12 mice | Two Way RM ANOVA  Bonferroni | WT: 0.131 ± 0.0233  *Gabra2*-1: 0.240 ± 0.0208 | 22 | genotype x LD/DD = 0.013 |

**Supplementary Table 7.** Antibodies used.

| **Antibody** | **Application** | **Dilution** | **Company / Source** | **Catalog** | **Authentication** |
| --- | --- | --- | --- | --- | --- |
| GABA_A_R α2 | Western blotting | 1:1000 | PhosphoSolutions | 822-A2CL | No 1^o^, KO |
| GABA_A_R α2 | Immuno | 1:500 | Synaptic Systems | 224 103 | No 1^o^, KO |
| GABA_A_R α2 | Immuno | 1:500 | Synaptic Systems | 224 104 | No 1^o^, KO |
| GABA_A_R α1 | Western blotting; Immuno | 1:500 | UC Davis/NIH NeuroMab | N95/35 | No 1^o^ |
| GABA_A_R α3 | Western blotting | 1:1000 | Synaptic Systems | 224 303 | No 1^o^ |
| Cb | Western blotting; Immuno | 1:500 | Synaptic Systems | 261 003 | No 1^o^, KO |
| NL2 | Western blotting | 1:1000 | Synaptic Systems | 129 205 | No 1^o^, KO |
| KCC2 | Western blotting | 1:1000 | Neuromab | 75-013 | No 1^o^ |
| NKCC1 | Western blotting | 1:1000 | Cell Signaling Technology | D13A9 #8351 | No 1^o^ |
| Syt2 | Immuno | 1:500 | Synaptic Systems | 105 225 | No 1^o^ |
| Parvalbumin | Immuno | 1:1000 | Sigma | P3088 | No 1^o^ |
| CB1R | Immuno | 1:500 | Synaptic Systems | 258 106 | No 1^o^ |
| Cholecystokinin | Immuno | 1:500 | abcam | ab37274 | No 1^o^ |
| Actin | Western blotting | 1:50000 | Millipore Sigma | A2228 | No 1^o^ |
